# Specific-detection of clinical samples, systematic functional investigations, and transcriptome analysis reveals that splice variant MUC4/Y contributes to the malignant progression of pancreatic cancer by triggering malignancy-related positive feedback loops signaling

**DOI:** 10.1186/s12967-014-0309-8

**Published:** 2014-11-04

**Authors:** Yi Zhu, Jing-Jing Zhang, Kun-Ling Xie, Jie Tang, Wen-Biao Liang, Rong Zhu, Yan Zhu, Bin Wang, Jin-Qiu Tao, Xiao-Fei Zhi, Zheng Li, Wen-Tao Gao, Kui-Rong Jiang, Yi Miao, Ze-Kuan Xu

**Affiliations:** Department of General Surgery, First Affiliated Hospital, Nanjing Medical University, 300 Guangzhou Road, Nanjing, 210029 Jiangsu Province People’s Republic of China; Jiangsu Province Academy of Clinical Medicine, Institute of Tumor Biology, Nanjing, 210029 People’s Republic of China; Jiangsu Province Blood Center, Nanjing, 210042 People’s Republic of China; Department of Pathology, Shanghai Medical College, Fudan University, Shanghai, 200032 People’s Republic of China; Department of Pathology, First Affiliated Hospital, Nanjing Medical University, Nanjing, 210029 People’s Republic of China; Department of General Surgery, the First Affiliated Hospital of Soochow University, Suzhou, 215006 People’s Republic of China

**Keywords:** MUC4/Y, Alternative splicing, Pancreatic neoplasms, Cell movement, Angiogenesis, Neoplasm metastasis, Gene expression regulation, Signal transduction

## Abstract

**Background:**

MUC4 plays important roles in the malignant progression of human pancreatic cancer. But the huge length of MUC4 gene fragment restricts its functional and mechanism research. As one of its splice variants, MUC4/Y with coding sequence is most similar to that of the full-length MUC4 (FL-MUC4), together with alternative splicing of the MUC4 transcript has been observed in pancreatic carcinomas but not in normal pancreas. So we speculated that MUC4/Y might be involved in malignant progression similarly to FL-MUC4, and as a research model of MUC4 in pancreatic cancer. The conjecture was confirmed in the present study.

**Methods:**

MUC4/Y expression was detected by real-time quantitative reverse transcription polymerase chain reaction (qRT-PCR) using gene-specific probe in the clinic samples. The effects of MUC4/Y were observed by serial *in vitro* and *in vivo* experiments based on stable over-expressed cell model. The underlying mechanisms were investigated by sequence-based transcriptome analysis and verified by qRT-PCR, Western blot and enzyme-linked immunosorbent assays.

**Results:**

The detection of clinical samples indicates that MUC4/Y is significantly positive-correlated with tumor invasion and distant metastases. Based on stable forced-expressed pancreatic cancer PANC-1 cell model, functional studies show that MUC4/Y enhances malignant activity *in vitro and in vivo*, including proliferation under low-nutritional-pressure, resistance to apoptosis, motility, invasiveness, angiogenesis, and distant metastasis. Mechanism studies indicate the novel finding that MUC4/Y triggers malignancy-related positive feedback loops for concomitantly up-regulating the expression of survival factors to resist adverse microenvironment and increasing the expression of an array of cytokines and adhesion molecules to affect the tumor milieu.

**Conclusions:**

In light of the enormity of the potential regulatory circuitry in cancer afforded by MUC4 and/or MUC4/Y, repressing MUC4 transcription, inhibiting post-transcriptional regulation, including alternative splicing, or blocking various pathways simultaneously may be helpful for controlling malignant progression. MUC4/Y- expression model is proven to a valuable tool for the further dissection of MUC4-mediated functions and mechanisms.

**Electronic supplementary material:**

The online version of this article (doi:10.1186/s12967-014-0309-8) contains supplementary material, which is available to authorized users.

## Background

Mucins are a family of high–molecular weight glycoproteins implicated in diverse biological function [1]. A member of the transmembrane mucin family [2,3], mucin 4 (MUC4) has been mapped to chromosome 3 in the q29 region, which was cloned from the human tracheobronchial chromosomal DNA library and a human pancreatic tumor cell line [2–5]. MUC4 plays important roles in the carcinogenesis and progression of multiple human cancers, including pancreatic cancer [6,7]. MUC4 is aberrantly expressed in pancreatic ductal adenocarcinoma (PDAC) and precancerous pancreatic intraepithelial neoplasias, but not in benign or normal parts of the pancreas [8–10], and the level of expression correlates significantly with poor prognosis of PDAC [10,11]. But because of the huge length of its gene fragment (more than 30 KB), it is incapable to clone and eukaryotic express the full-length MUC4 gene. The function predictions and structure analyses of human MUC4 were mostly from the overexpression of its rat homologous gene rMuc4/SMC on human tumor cells.

We have noticed that alternative splicing of the MUC4 transcript has been observed in pancreatic carcinomas but not in normal pancreas [12–14]. MUC4 has a series of splice variants (SV), including an SV1-21 isoform termed SV0 (the full-length MUC4 [FL-MUC4]), MUC4/X, and MUC4/Y. MUC4/Y has the coding sequence most similar to that of FL-MUC4 [15]. Though it lacks the coding exon 2, MUC4/Y has the same exon sequences as FL-MUC4, and the encoded protein has the same conserved domains and a transmembrane β-subunit that contains three epidermal growth factor (EGF) domains, a transmembrane sequence, a short cytoplasmic tail, and multiple *N*-glycosylation sites as those in FL-MUC4. So we speculated that MUC4/Y may be involved in carcinogenesis and progression similarly to FL-MUC4. More importantly, it may be as a research function model of MUC4 in pancreatic cancer. Furthermore, based on MUC4/Y, it is easy to construct the unique domain-lacking models of MUC4 for its structure analyses and mechanisms dissection.

Thus, the present study aimed to illustrate the role of MUC4/Y in the progression of pancreatic cancer and give insights into its downstream signaling effects: 1) Quantitatively assess MUC4/Y expression in tissue samples from PDAC patients using probe-specific primers were done to illustrate the correlation of MUC4/Y expression with clinicopathological factors and PDAC patient survival. 2)Various *in vitro* and *in vivo* assays were conducted using stable MUC4/Y-overexpressing pancreatic cancer cell models to illustrate its function in promoting the malignant properties of pancreatic cancer. 3) The underlying molecular mechanisms were investigated by sequence-based digital gene expression (DGE) analysis and bioinformatics analyses to provide full explanations.

## Methods

### Selection of patients and tissue specimens

We enrolled 108 patients for this retrospective study. The patients had undergone pancreaticoduodenectomy (Whipple resection) with histologically proven PDAC at the Department of General Surgery of the First Affiliated Hospital of Nanjing Medical University between 2006 and 2012. The Ethics Committee of the First Affiliated Hospital of Nanjing Medical University (Permit Number: 2009-SR-031) approved this study. Each patient provided informed consent.

The patients (61 men and 47 women; age range: 25–82 years; mean age: 60.98 ± 11.39 years) were regularly followed until May 31, 2013. Overall survival (OS) was defined as the time between surgery and death or the last follow-up date. No patient died within one month after surgery. Additional file 1: Table S1 summarizes the corresponding characteristics of the patients; staging and grading were based on the sixth edition of the American Joint Committee on Cancer guidelines [16].

Tissue samples were removed as soon as possible after resection and divided into at least two bulk tissue samples. A part of each sample was snap-frozen in liquid nitrogen and then stored in liquid nitrogen until used for RNA extraction. The remainder was fixed in formalin, embedded in paraffin, and cut into 4-μm thick sections for hematoxylin–eosin (H&E) staining. All tissue samples were examined histologically; experienced pathologists confirmed the diagnosis. Histological grades of tumor differentiation were assigned according to World Health Organization criteria.

### RNA extraction and quantitative assessment of MUC4/Y and MUC4 by real-time reverse transcription–PCR with specific primers

**Figure 1:**
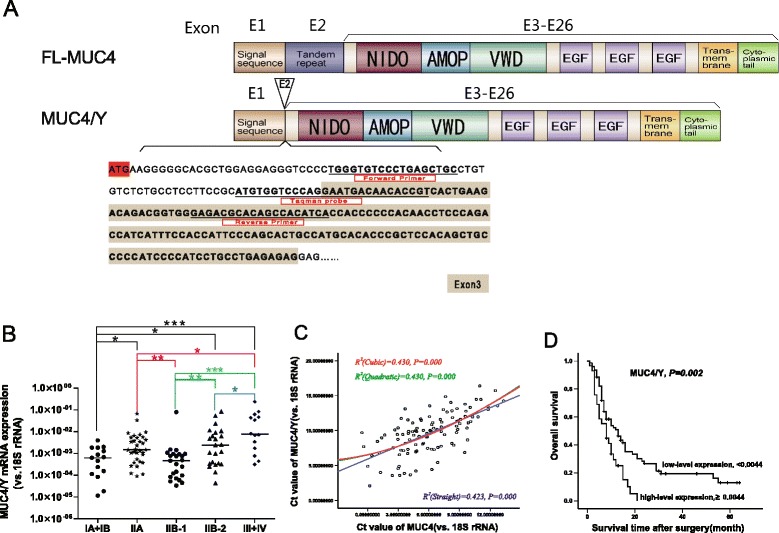
**Significant positive correlation between MUC4/Y mRNA expression level and TNM stage, MUC4 mRNA expression level, and survival in PDAC. (A)** Schematic representation of the design strategy for specific primers and TaqMan probe in MUC4/Y gene detection based on the difference between the exon sequences of MUC4/Y (NCBI Reference Sequence: NM_004532.5) and FL-MUC4 (NCBI Reference Sequence: NM_018406.6). The TaqMan probe sequence lies in the exon 1–exon 3 junction; as exon 2 is absent, it detects MUC4/Y expression rather than other MUC4 types encoding the exon 1–exon 2 junction, or MUC4/X (NCBI Reference Sequence: NM_138297.4), which encodes the exon 1–exon 4 junction because it lacks the coding exons 2 and 3. Primers and TaqMan probe are underlined. **(B)** Comparison of MUC4/Y mRNA expression at different TNM stages. Scatter dot plots were drawn from the minimum extending to the maximum; the center horizontal line denotes the sample median. *P <0.05, **P ≤0.01, ***P ≤0.001. **(C)** Positive correlation between MUC4/Y and MUC4 mRNA expression (*R* ^*2*^ = 0.430, *P* <0.001) and curve fitting. Regression equations were Y = 6.553 + 0.3212 × X + 0.02719 × X/2–0.00033333 × X/3 (cubic), Y = 6.522 + 0.3514 × X + 0.02102 × X/2 (quadratic), and Y = 5.915 + 0.6127 × X (straight line). According to the correlation coefficient value, the cubic or quadratic curve model was the better model. Ct, threshold cycle value. **(D)** Kaplan–Meier survival curves of PDAC patients according to MUC4/Y mRNA expression status. The *P*-value was calculated using the log-rank test.

Following liquid nitrogen grinding, total RNA was extracted from bulk tissues with TRIzol (Life Technologies) according to the manufacturer’s protocol. After spectrophotometry quantification, 1 μg total RNA was used in a final volume of 20 μL for reverse transcription (RT) with an iScript cDNA Synthesis Kit ((Bio-Rad, CA, USA) according to the manufacturer’s instructions. Quantitative real-time PCR was performed using TaqMan Gene Expression Assays (Life Technologies) in a StepOnePlus Real-Time PCR System (Life Technologies). Reactions were performed in 10-μL volumes containing 1 μL diluted complementary DNA (cDNA), 20× TaqMan Gene Expression Assay Mix, and 2× TaqMan Universal PCR Master Mix. The thermal cycling conditions comprised initial denaturation at 95°C for 10 min and 40 cycles at 95°C for 15 s and 60°C for 1 min. The product number of the MUC4 TaqMan Gene Expression Assay Mix was Hs003666414 (Applied Biosystems). Figure 1A depicts the specific primers (forward: 5′-TGGGTGTCCCTGAGCTGC-3′, reverse: 5′-TGATGTGGCTGTGCGTCTC-3′) and TaqMan probe (5′-ATGTGGTCCCAGGAATGACAACACCGT-3′) designed for MUC4/Y. In addition to BLASTN searches, we performed RT-PCR with human pancreatic cancer HPAC cell-lines using the MUC4/Y forward and reverse primers, and the target PCR product was subcloned into a pMD18-T vector for DNA sequencing to ensure the specificity of each primer and confirm that the sequence was correct. Human 18S rRNA (Hs099999901_s1; Applied Biosystems) was used as the internal control for each sample to calibrate the original concentration of mRNA [17]. Relative gene expression was calculated by subtracting the threshold cycle (Ct) value of the target genes and 18S rRNA (control) genes using the 2^-ΔCt^ method [18]. Each quantification PCR was performed in triplicate and repeated thrice independently.

### Cell culture and stable overexpression of MUC4/Y

The PANC-1 pancreatic cancer cell line was obtained from the Shanghai Institutes for Biological Sciences, Chinese Academy of Sciences, and grown in Dulbecco’s modified Eagle’s medium (DMEM) containing high glucose (Wisent Biocenter, Canada) and supplemented with 10% (v/v) fetal bovine serum (FBS; Wisent Biocenter, Canada) and penicillin–streptomycin (HyClone, Thermo, USA) at 37°C in a humidified atmosphere containing 5% CO_2_.

We designed full-length cDNA fragments encoding the MUC4/Y gene, i.e., human MUC4 cell surface–associated transcript variant 4 (NCBI Reference Sequence: NM_004532.5, GI:336285420) containing a Kozak sequence (GCCACC) before the ATG initiation codon for optimal translation and with unique restriction sites present in the multiple clone site (MCS) of the lentiviral vector but absent from the MUC4/Y cDNA sequence. The target sequence was synthesized and cloned in a pUC57 vector (GenScript). Then, the cDNA encoding MUC4/Y was subcloned into the lentiviral vector pCDH-CMV-MCS-EF1-Puro (Cat. #CD510B-1, System Biosciences, USA). To generate viral particles, 293 T cells were transiently transfected with pCDH-CMV-MCS-EF1-Puro/MUC4/Y and the pPACKH1 Lentivector Packaging Kit (Cat. #LV500A-1, System Biosciences, USA) with Lipofectamine 2000 (Invitrogen Life Technology) according to the manufacturer’s instructions. The virus titer was detected with quantitative real-time PCR after concentrating and harvesting the viral supernatant 48 h after transfection. At 24 h after plating, stable transduction of PANC-1 cells was carried out at 20 multiplicity of infection with the virus and using polybrene (8 μg/mL; Sigma-Aldrich) to augment infection efficiency. Stable clones were then selected in medium containing puromycin (2 μg/mL; Sigma-Aldrich).

Using real-time PCR, western blotting, and immunofluorescence, we detected the mRNA and protein expression and location of the target genes. Stable transfected PANC-1 cells overexpressing the *MUC4/Y* gene were designated PANC-1-MUC4/Y; PANC-1 cells transfected with empty lentiviral vectors (EV) were designated PANC-1-EV. Wild-type PANC-1 and PANC-1-EV cells were used as blank and negative control groups, respectively. No differences were observed between the PANC-1-EV and wild-type PANC-1 cells.

### Western blotting

PANC-1–derived clones were processed for protein extraction and western blotting using standard procedures. Cell lysates were prepared as described previously [19]. After the concentrations were determined using the Bradford assay, proteins (30 μg/lane) were resolved on 4-20% Mini-PROTEAN TGX precast gels (#456-1093; Bio-Rad). The resolved proteins were transferred onto polyvinylidene difluoride membranes, blocked with 5% non-fat milk in phosphate-buffered saline (PBS) for 2 h, and subjected to standard immunodetection procedures using specific antibodies. The following primary antibodies were used to label the membranes: 1 μg/mL anti-MUC4 mouse monoclonal antibody (ab60720; Abcam, Cambridge, UK) and anti–glyceraldehyde-3-phosphate dehydrogenase (GAPDH) mouse monoclonal antibody (1:1000, AG019; Beyotime, CHINA). The membranes were incubated at 4°C overnight, followed by six 10-min washes in TBST [50 mmol/L Tris–HCl (pH 7.4), 150 mmol/L NaCl, 0.05% Tween 20], and were incubated with horseradish peroxidase–labeled goat anti-mouse immunoglobulin G (IgG, 1:2000; #A0216; Beyotime) for 1 h at room temperature, followed by six 10-min washes with TBST. The blots were developed with an enhanced chemiluminescence kit (Amersham, Freiburg, Germany). The internal molecular weight standard was PageRuler Plus Prestained Protein Ladder (#26619/SM1811, 10–250 kD; Fermentas).

### Immunofluorescence

Cells cultured to 70% confluence were washed with 0.1 mol/L HEPES containing Hanks’ buffer and fixed with Immunol Staining Fix Solution (#P0098; Beyotime) at room temperature for 15 min. After three 10-min washes in Immunol Staining Wash Buffer (#P0106; Beyotime), the fixed cells were blocked in Immunol Staining Blocking Buffer (#P0102; Beyotime) for 60 min at room temperature for nonspecific blocking, followed by incubation at 4°C overnight with anti-MUC4 mouse monoclonal antibody (1:100 in PBS, ab60720; Abcam). Cells were washed 3–5 times for 10 min with PBS containing 0.05% Tween 20 (PBS-T) and then incubated with cyanine 3–labeled goat anti-mouse secondary antibodies (#P0193; Beyotime) at 37°C for 60 min. The cells were washed again 3–5 times for 10 min with PBS-T. Nuclei were counterstained with diaminophenylindole (0.5 μg/mL) for 5 minutes. Immunostaining was observed under a Nikon Ti-E inverted fluorescence microscope; representative photographs were captured using NIS-Elements D4.0 software (Nikon).

### Cell proliferation assay

Cell proliferation was determined using Cell Counting Kit-8 (#C0038; Beyotime) according to the manufacturer’s instructions. Briefly, 1 × 10^3^ cells/well were seeded in a 96-well flat-bottomed plate and grown at 37°C for 24, 48, 72, 96, and 120 h. Cells were grown in high- (10% FBS) or low-serum (1% FBS) medium. After 10 μL WST-8 dye was added to each well, cells were incubated at 37°C for 4 h and the absorbance at 450 nm determined using a microplate reader.

### Apoptosis assay

Many agents, including the clinically useful sorafenib, can induce apoptosis [20]. Following 24-h treatment with 8 μM sorafenib, PANC-1–derived clones were trypsinized, washed with cold PBS, and resuspended in PBS. The advantage of 7-amino-actinomycin D (7-AAD) over propidium iodide is that there is minimal spectral overlap between the emissions. We used a fluorescein isothiocyanate (FITC) Annexin V Apoptosis Detection Kit with 7-AAD (#640922; BioLegend, USA) according to the manufacturer’s instructions. Briefly, 5 μL FITC–annexin V and 5 μL 7-AAD Viability Staining Solution (#00-6993; eBioscience, USA) were added to a 100-μL cell suspension (1 × 10^6^ cells) in binding buffer. The cells were gently vortexed and incubated for 15 min at room temperature in the dark, and 400 μL binding buffer was added for flow cytometric analysis using a FACScan Flow Cytometer (Becton Dickinson).

### Cell migration and invasion assays

We used modified 24-well Boyden chambers for the cell migration and invasion assays. The top chamber (Transwell) containing a polycarbonate filter membrane (8-μm pore size; BD Labware) was inserted into a 24-well plate (bottom chamber); a Transwell filter membrane coated with 40 μL Matrigel (BD Biosciences) was used in the invasion assay. Medium containing 10% FBS was placed in the bottom chamber as a chemoattractant. Cells (3 × 10^5^) in 300 μL serum-free medium were placed in the top chamber and incubated at 37°C for 24 h. Cells that had migrated or invaded through the Matrigel on the bottom surface of the filter were fixed with 4% paraformaldehyde for 10 min and stained with 5% crystal violet (Sigma-Aldrich) in 25% methanol for 10 min. Cells on the top surface of the filter (cells that did not migrate or invade through the Matrigel) were removed using a moist cotton swab. Cells in 10 random fields were evaluated under × 400 magnification. The experiments were repeated thrice. Data were expressed as the number of cells per area.

### Endotube formation assay

We measured endotube formation by human umbilical vein endothelial cells (HUVECs) using an angiogenesis assay on Matrigel (Growth Factor Reduced Matrigel Matrix; BD Biosciences). To investigate the influence of MUC4/Y overexpression on PANC-1 cell–HUVEC interaction on HUVEC endotube formation, we co-cultured HUVECs with control and genetically modified PANC-1 cells using a double-chamber method in 24-well tissue culture plates. Stable cell lines (1 × 10^5^ cells) were seeded into Transwell chambers consisting of polycarbonate membranes with 0.4-mm pores (BD Biosciences) and allowed to adhere overnight. To reconstitute the basement membrane, Matrigel was diluted 2-fold with cold DMEM (without FBS) and added to the plates (250 μL/well) at 4°C. Plates were incubated for 2 h in a 37°C cell culture incubator to allow the Matrigel to solidify. HUVECs were trypsinized, counted, resuspended in basal medium, and added on top of the reconstituted basement membrane (5 × 10^4^ cells/well). The top chamber was then placed in the HUVEC endotube formation assay system on Matrigel as described above. Cells were incubated for 16 h to allow tube formation. Endotubes were quantified by counting five random fields from each sample under × 40 magnification.

### Animals

Thirty-two female BALB/C nude mice were purchased from the Nanjing University Model Animal Research Center and housed in specific pathogen–free conditions. This study was conducted in strict accordance with the recommendations in the Guide for the Care and Use of Laboratory Animals of the Ministry of Health, China. The Ethics Committee of the First Affiliated Hospital of Nanjing Medical University (Permit Number: 2012-SRFA-093) approved the protocol. The mice were randomly assigned to four groups when they were 3–5 weeks old and weighed 18–20 g: two groups each for subcutaneous and orthotopic models. All surgery was conducted under sodium pentobarbital anesthesia and all efforts were made to minimize suffering.

### Subcutaneous model and bioluminescence imaging

PANC-1–derived clones, i.e., PANC-1-MUC4/Y and PANC-1-EV, were re-transfected with lentivirus (#pSB72; ShangHai SBO Medical Biotechnology, CHINA) expressing both luciferase and green fluorescent protein (GFP). GFP-expressing cells were sorted using a fluorescence-activated cell sorter (FACS) and termed PANC-1-MUC4/Y-Luc and PANC-1-EV-Luc (negative control), respectively. Subconfluent cultures of PANC-1-MUC4/Y-Luc and PANC-1-EV-Luc cells were trypsinized and washed with PBS. Cell viability was determined by trypan blue staining; single-cell suspensions with >90% viability were used for the subcutaneous injections. Cells in 100-μL suspensions (1 × 10^7^) from each group of cells were injected subcutaneously into the flanks of the animals. Beetle luciferin (150 mg/kg in PBS; Promega) was used as the substrate for the luciferase-expressing cells and injected intravenously 1–2 minutes before imaging. Mice were anesthetized using 3.5% chloral hydrate and imaged at 2 hours and 3, 10, 15, 21, 26, 30 days after tumor cell injection and using a cooled charged-coupled device camera (IVIS system; Xenogen). Exposure times for saturated images were reduced accordingly. Images were quantified as photons/s using Living Image software (Xenogen). *In vivo* tumor growth was monitored by measuring bioluminescence imaging (BLI). At 30 days after implantation, the mice were sacrificed and the tumors harvested for paraffin embedding and H&E staining, and immunohistochemical (IHC) analysis.

### IHC, Ki67 staining, TUNEL, microvessel density assay

IHC staining was carried out using the standard avidin-biotin complex method using Ki67 antibody (Dako, MIB-1, 1:200). Immune reactions were visualized with 3,3-diaminobenzidine and counterstained with Mayer’s hematoxylin. Ki67-positive cells were counted and presented as the average of the five highest areas within a single × 400 magnification field. Terminal deoxynucleotidyl transferase–mediated dUTP nick end labeling (TUNEL) was carried out using an ApopTag Plus Peroxidase In Situ Apoptosis Detection kit (Intergen) according to the manufacturer’s instructions. Briefly, slides were deparaffinized and treated with 20 μg/mL proteinase K at 37°C for 15 min to enhance staining. After immersion in 3% hydrogen peroxide to block endogenous peroxidase, slides were incubated with reaction buffer containing terminal deoxynucleotidyl transferase at 37°C for 1 h. The slides were then incubated with peroxidase-conjugated anti-digoxigenin antibody for 30 min, and the reaction products were visualized with 0.03% 3,3-diaminobenzidine containing 2 mmol/L hydrogen peroxide. TUNEL-positive cells were counted and presented as the average of the five highest areas within a single × 200 field. To determine microvessel density (MVD), 5-μm paraffin-embedded sections were stained with rat anti-mouse CD31 monoclonal antibody (BD Biosciences). The average number of CD31-positive vessels per field, denoting MVD, were examined under × 100 magnification and counted.

### Orthotopic model metastasis assay

Cells (2 × 10^6^, 50-μL suspensions) from PANC-1–derived cell lines were orthotopically implanted in the pancreas of nude mice as previously described [21]. The mice were sacrificed at 45 days after implantation. The presence of metastatic lesions in other organs was determined by thorough gross inspection and histological analysis.

### Sequence-based digital gene expression analysis

Total RNA was extracted from three groups of cells: PANC-1-MUC4/Y, wild-type PANC-1, and PANC-1-EV using TRIzol (Invitrogen) according to the manufacturer’s protocol. RNA integrity was confirmed using a 2100 Bioanalyzer (Agilent Technologies). The samples intended for transcriptome analysis were processed by BGI. Briefly, 6 μg total RNA was extracted, the mRNA purified using oligo(dT) magnetic bead adsorption, and cDNA synthesized using oligo(dT) as the primer. The 5′ ends of tags can be generated using two endonucleases: *Nla*III or *Dpn*II. Typically, the bead-bound cDNA is digested with *Nla*III, which recognizes and cuts off CATG sites. Fragments other than the 3′ cDNA fragments connected to the oligo(dT) beads are washed away and Illumina adaptor 1 is ligated to the sticky 5′ end of the digested, bead-bound cDNA fragments. The junction of adaptor 1 and a CATG site is the recognition site for *Mme*I, an endonuclease with separate recognition and digestion sites. It cuts 17 bp downstream of the CATG site, producing tags with adaptor 1. After removing the 3′ fragments with magnetic bead precipitation, Illumina adaptor 2 is ligated to the 3′ ends of the tags, producing tags with different adaptors at both ends to form a tag library. Following linear PCR amplification, fragments are purified by polyacrylamide gel electrophoresis. During the quality control steps, the Agilent 2100 Bioanalyzer and Applied Biosystems StepOnePlus Real-Time PCR System are used to quantify and qualify the sample library, which is sequenced using Illumina HiSeq 2000. The raw tag sequence data are analyzed for gene annotation and normalization, screening of differentially expressed genes (DEGs), and functional annotation through the in-house bioinformatics analysis pipeline.

### DEG Gene Ontology functional enrichment and pathway enrichment analysis

DEGs annotated against the Gene Ontology (GO) and Kyoto Encyclopedia of Genes and Genomes (KEGG) databases were enriched to identify significant GO biological process terms and pathways, respectively, and adjusted with corrected *P* ≤0.05 for GO analysis and pathways.

### Verification of DEGs and VEGF family molecule**s**

Additional file 1: Table S2 lists the sequences of the specific primer sets. The endogenous control gene (18S rRNA) was used as an internal control. Quantitative RT-PCR (qRT-PCR) was performed using a SYBR Premix Ex *Taq* Kit (TaKaRa) according to the manufacturer’s protocol. A no-template control sample (nuclease-free water) was included to detect contamination and to determine the degree of dimer formation. Ct values were normalized to the *18S* gene and a relative quantitative method (ΔΔCt) was used to evaluate quantitative variation. To determine biological variability within cell lines, we measured up to three independent RNA samples per line. cDNA was generated using an iScript cDNA Synthesis Kit (Bio-Rad).

Additional file 1: Table S3 lists the sets of specific antibodies and concentration used; we performed Western blotting assays as described above.

To confirm the mechanism of MUC4/Y enhancement of HUVEC endotube formation, we performed enzyme-linked immunosorbent assay (ELISA) of vascular endothelial growth factor (VEGF, main detection of VEGFA) and interleukin-8 (IL8) in cell culture supernatants using a kit as per the manufacturer’s instructions (R&D Systems). We collected the supernatant from PANC-1-EV, and PANC-1-MUC4/Y cell cultures. Cells (1 × 10^5^/mL) were seeded in a 24-well plate and cultured overnight. The medium was replaced and cells were cultured for another 24 h or 48 h. The culture media were collected and microfuged at 1500 rpm for 5 min to remove particles, and the supernatant stored at −80°C until used in the ELISA.

### Statistical analysis

Statistical analysis was performed using SPSS 17.0 (SPSS Inc.). The results were confirmed by conducting at least three independent experiments for all *in vitro* and *in vivo* experiments.

Data obtained from patients and tissue specimens were analyzed as described previously [10]. Data were analyzed using the Mann–Whitney *U* test (or Kruskal–Wallis test). All statistical tests were 2-tailed exact tests, with *P* <0.05 considered significant. Based on MUC4/Y expression level cut-off values determined by receiver operating characteristic (ROC) curve analysis, survival distributions were estimated using the Kaplan–Meier method. Variables with statistically significant prognostic value in univariate analyses were entered into a multivariate model and excluded where *P* >0.10. Stepwise selection of factors was applied to the multivariate Cox regression model to identify independent prognostic factors for OS.

Between the two groups in the subcutaneous model, the mean tumor growth rates of Luc-PANC-1 cells at different time points determined by BLI (photons/s) were compared using repeated-measures analysis of variance (ANOVA) to identify subject-by-time profiles.

Between the two groups in the orthotopic model, incidence of metastasis was compared using Fisher’s exact test.

All data presented are the mean ± standard deviation (SD) of *n* independent measurements unless noted otherwise. Statistical analysis was performed with one-way ANOVA for multiple groups and the unpaired Student *t*-test for individual groups; statistical significance was assigned when *P* <0.05.

## Results

### MUC4/Y mRNA expression was significantly positively correlated with tumor invasion, distant metastases and MUC4 mRNA expression level

Figure 1 depicts the level of MUC4/Y expression normalized to that of 18S rRNA. As shown in Table 1, The Mann–Whitney *U* test (2-tailed exact tests) demonstrated the level of tumor MUC4/Y expression was significantly correlated with tumor-node-metastasis (TNM) stage (*P* = 0.001), but no significant correlations were identified between the levels of tumor *MUC4/Y* expression and other variables. TNM Staging System for pancreatic cancer were shown in Additional file 1: Table S1. Here Stage IIB can be divided in two groups: one defined as IIB-1, i.e. Tumor limited to the pancreas, Regional lymph node metastasis, No distantant metastasis (T1 + T2, N1, M0); the other defined as IIB-2, i.e. Tumor extends beyond pancreas but locally invasive, Regional lymph node metastasis, No distantant metastasis (T3, N1, M0). As shown in Figure 1B, MUC4/Y expression at TNM stage IA + IB was significantly lower than that at TNM stage IIA, IIB-2 or III + IV (*P* = 0.012, 0.022, 0.001). MUC4/Y expression at TNM stage IIA was significantly lower than that at TNM stage III + IV (*P* = 0.012). MUC4/Y expression at TNM stage IIB-1 was significantly lower than that at TNM stage IIA, IIB-2 or III + IV (*P* = 0.004, 0.004, 0.000). MUC4/Y expression at TNM stage IIB-2 was significantly lower than that at TNM stage III + IV (*P* = 0.047). There was no significant difference between MUC4/Y expression at TNM stage IA + IB and IIB-1(*P* = 0.591). There was also no significant difference between MUC4/Y expression at TNM stage IIA and IIB-2 (*P* = 0.752).

**Table 1:** **Clinicopathological factors and the expression of MUC4/Y in 108 patients with PDAC**

**Category**	**N(%)**	**MUC4/Y**		
		**Mean**	**Median(range)**	***P*** **Value**
Age(y)				
<60	47 (43.52)	0.0076	0.0010(0.0000-0.0829)	0.689
≥60	61 (56.48)	0.0112	0.0012(0.0000-0.2350)	
Gender				
Male	61 (56.48)	0.0117	0.0013(0.0000-0.2350)	0.228
Female	47 (43.52)	0.0070	0.0010(0.0000-0.0787)	
Location of tumor				
Head	72 (66.67)	0.0071	0.0010(0.0000-0.0842)	0.494
Body and tail	36 (33.33)	0.0147	0.0015(0.0000-0.2350)	
Size of tumor				
≤2 cm	22 (20.37)	0.0074	0.0010(0.0000-0.0682)	0.647
>2 cm	86 (79.63)	0.0102	0.0011(0.0000-0.2350)	
Nerve infiltration				
No	38 (35.19)	0.0098	0.0007(0.0000-0.0842)	0.137
Yes	70 (64.81)	0.0096	0.0012(0.0000-0.2350)	
Differentiation				
Well	15 (13.89)	0.0267	0.0012(0.0000-0.2350)	0.979
Moderate	83 (76.85)	0.0071	0.0011(0.0000-0.0842)	
Poor	10 (9.26)	0.0055	0.0011(0.0001-0.0340)	
TNM^a^ staging				
IA + IB	15 (13.89)	0.0009	0.0006(0.0000-0.0040)	0.001*
IIA	33 (30.56)	0.0056	0.0015(0.0001-0.0682)	
IIB	46 (42.59)	0.0069	0.0008(0.0000-0.0842)	
III + IV	14 (12.96)	0.0380	0.0076(0.0003-0.2350)	
Serum CA19-9^b^ level				
≤39KU/l	31 (28.70)	0.0155	0.0009(0.0000-0.2350)	0.965
>39KU/l	77 (71.30)	0.0073	0.0011(0.0000-0.8423)	
Serum CA50^c^ level				
≤25KU/l	49 (45.37)	0.0152	0.0009 (0.0000-0.2350)	0.894
>25KU/l	59 (54.63)	0.0050	0.0011(0.0000-0.0829)	
Serum CEA^d^ level				
≤5 μg/l	64 (59.26)	0.0093	0.0010(0.0000-0.0842)	0.557
>5 μg/l	44 (40.74)	0.0101	0.0011(0.0000-0.2350)	

TNM^a^, tumor-node-metastasis; CA19-9^b^, carbohydrate antigen 19–9; CA50^c^, carbohydrate antigen 50; CEA^d^, carcinoembryonic antigen; *P <0.05.

We quantified the correlation between MUC4/Y and MUC4 expression levels in pancreatic cancer tissues with real-time PCR. Figure 1C depicts a positive correlation between MUC4/Y and MUC4 expression levels in PDAC (*R*^*2*^ = 0.430, *P* <0.001).

### Association between MUC4/Y mRNA expression levels, or clinicopathological factors and PDAC patient survival

We enrolled 108 PDAC patients in the survival analysis. Eighty-four patients died; the remaining 24 patients were alive at the last follow-up (May 31, 2013). The OS rates at 12, 18, and 24 months were 37.96%, 21.30%, and 15.74%, respectively. Patients were divided into two groups based on the length of OS: short-term survivors (survival <24 months) and long-term survivors (survival ≥24 months).

The threshold value of 0.0044 was chosen as the cut-off score for both high and low MUC4/Y expression, as 0.0044 (within the MUC4/Y expression 95% confidence interval [CI] of 0.0043-0.0150) was on the ROC curve closest to (0.0, 1.0). This maximized both sensitivity and specificity for survival outcome. The area under the ROC curve was 0.861 (95% CI: 0.784-0.938, *P* = 0.000).

The Kaplan–Meier survival curves showed that postoperative survival was shorter for patients with high (≥0.0044) MUC4/Y expression compared to patients with low (<0.0044) expression (*P* = 0.002, log rank test; Figure 1D).

**Figure 2:**
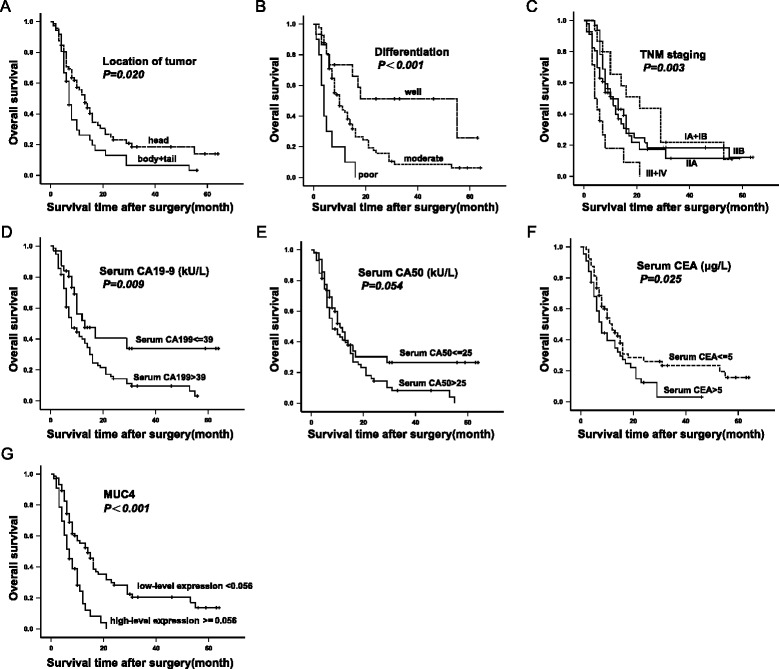
**Kaplan-Meier survival estimate by clinicopathological factors for 108 PDAC patients who underwent pancreaticoduodenectomy and survived at least 30 days after surgery.** The *p* value was calculated by the Log-rank test. The correlation between survival and tumor location **(A)**, tumor differentiation **(B)**, TNM staging **(C)**, serum level of CA19-9 **(D)**, serum level of CA50 **(E)**, serum level of CEA **(F)**, MUC4 mRNA expression status **(G)**, respectively, among which the *P* value of serum level of CA50 lied in the critical (*P = 0.054)*.

As shown in Figure 2, the survival of patients with tumors occurring in the body or tail of the pancreas, poor tumor differentiation, TNM staging of III + IV, high serum (>39 kU/L) of CA19-9, high serum (>5 μg/L) of CEA, high (≥0.056) MUC4/Y expression was significantly worse than those with tumors occurring in the head of the pancreas*(P = 0.020*, log rank test), well and moderate tumor differentiation *(P<0.001,* log rank test), TNM staging of A + IB or IIA or IIB *(P = 0.003*, log rank test), low serum level of CA19-9*(P = 0.009*, log rank test), low serum level of CEA*(P = 0.025*, log rank test), low MUC4/Y expression*(P<0.001*, log rank test), respectively. There were no significant associations between survival and patient gender, age, tumor size, nerve infiltration (*P = 0.489, 0.173, 0.340, 0.689*, respectively; log rank test). The log rank test showed the *P* value of serum level of CA50 lied in the critical (*P = 0.054)*.

Using *P* = 0.10 as the cut-off value, eight factors (tumor location, tumor differentiation, TNM stage, serum carbohydrate antigen [CA]19-9, serum CA50, serum carcinoembryonic antigen, MUC4 expression, and MUC4/Y expression) were selected from the univariate analysis data (Table 2) for forward or backward stepwise multivariate Cox proportional hazard analysis (Table 3). Forward stepwise multivariate Cox analysis determined that tumor differentiation (*P* = 0.001; hazard ratio [HR], moderate: 2.368, 95% CI: 1.120-5.007; HR, poor: 6.603, 95% CI: 2.515-17.338), serum CA19-9 (*P* = 0.021; HR: 1.914, 95% CI: 1.105-3.318), and MUC4 expression (*P* = 0.001; HR: 2.281, 95% CI: 1.415-3.677) were significant independent risk factors. Backward stepwise multivariate Cox analysis determined that tumor location (*P* = 0.001; HR: 2.452, 95% CI: 1.414-4.253), tumor differentiation (*P* <0.001; HR, moderate: 2.163, 95% CI: 0.994-4.706; HR, poor: 7.076, 95% CI: 2.655-18.857), TNM stage (*P* = 0.014; HR, IIA: 3.395, 95% CI: 1.414-8.148; HR, IIB: 3.551, 95% CI: 1.523-8.277; HR, III + IV: 4.218, 95% CI: 1.652-10.768), serum CA19-9 (*P* = 0.008; HR: 2.817, 95% CI: 1.313-6.043), and MUC4 expression (*P* = 0.010; HR: 1.976, 95% CI: 1.174-3.326) were significant independent risk factors.

**Table 2:** **Univariate analysis of prognostic factors**

**Variable**	**Cases**	**Events**	**Mean survival (months)**	**HR** ^**a**^ ** (95% CI** ^**b**^ **)**	***p***
Gender					0.504
Male	61	47	16.176	1	
Female	47	37	19.699	0.862 (0.559-1.331)	
Age (y)					0.176
< 60	47	35	21.381	1	
≥ 60	61	49	15.254	1.352 (0.874-2.092)	
Location of tumor					0.026^*^
Head	72	52	20.770	1	
Body and tail	36	32	12.235	1.655 (1.062-2.579)	
Size of tumor (cm)					0.358
≤ 2	22	67	20.116	1	
>2	86	17	17.290	1.285 (0.753-2.192)	
Differentiation					<0.001^*^
Well	15	8	34.953	1	
Moderate	83	66	15.803	2.532 (1.202-5.332)	0.015^*^
Poor	10	10	5.700	7.624 (2.918-19.916)	<0.001^*^
Nerve infiltration					0.699
No	38	29	21.137	1	
Yes	70	55	16.406	1.094 (0.694-1.725)	
TNM staging					0.007^*^
IA + IB	15	12	24.448	1	
IIA	33	25	17.483	1.371 (0.684-2.751)	0.374
IIB	46	34	18.785	1.507 (0.777-2.925)	0.225
III + IV	14	13	6.982	3.729 (1.664-8.358))	0.001^*^
Serum CA19-9 (kU/L)					0.014^*^
≤ 39	31	16	28.993	1	
> 39	77	68	14.556	1.988 (1.150-3.436)	
Serum CA50 (kU/L)					0.065^*^
≤ 25	49	30	24.091	1	
> 25	59	54	14.459	1.529 (0.975-2.398)	
Serum CEA (μg/L)					0.032^*^
≤ 5	64	44	22.116	1	
> 5	44	40	12.072	1.609 (1.041-2.488)	
MUC4 (2^-ΔCt^)					<0.001^*^
Low (<0.056)	75	54	22.101	1	
High (≥0.056)	33	30	8.054	2.595 (1.612-4.179)	
MUC4/Y (2^-ΔCt^)					0.003^*^
Low (<0.0044)	79	58	21.126	1	
High (≥0.0044)	29	26	8.835	2.077 (1.283-3.365)	

^a^HR, hazard ratio; ^b^CI, confidence interval; ^*^ *P ≤* 0.10.

**Table 3:** **Multivariate analysis of prognostic factors**

**Variable**	**Forward stepwise**		**Backward stepwise**	
	**HR** ^**a**^ **(95% CI** ^**b**^ **)**	***p***	**HR (95% CI)**	***p***
Location of tumor		0.085		0.001^*^
Head	-		1	
Body and tail	-		2.452 (1.414-4.253)	
Differentiation		0.001^*^		<0.001^*^
Well	1		1	
Moderate	2.368 (1.120-5.007)	0.024^*^	2.163 (0.994-4.706)	0.052
Poor	6.603 (2.515-17.338)	<0.001^*^	7.076 (2.655-18.857)	<0.001^*^
TNM staging		0.175		0.014^*^
IA + IB	-		1	
IIA	-	0.929	3.395 (1.414-8.148)	0.006^*^
IIB	-	0.922	3.551 (1.523-8.277)	0.003^*^
III + IV	-	0.077	4.218 (1.652-10.768)	0.003^*^
Serum CA19-9 (kU/L)		0.021^*^		0.008^*^
≤ 39	1		1	
> 39	1.914 (1.105-3.318)		2.817 (1.313-6.043)	
Serum CA50 (kU/L)		0.535		0.085
≤ 25	-		1	
> 25	-		0.552 (0.280-1.086)	
Serum CEA (μg/L)		0.265		0.089
≤ 4.3	-		1	
> 4.3	-		1.554 (0.934-2.584)	
MUC4 (2^-ΔCt^)		0.001^*^		0.010^*^
Low (<0.056)	1		1	
High (≥0.056)	2.281 (1.415-3.677)		1.976 (1.174-3.326)	
MUC4/Y (2^-ΔCt^)		0.067		0.243
Low (<0.0044)	-		-	
High (≥0.0044)	-		-	

^a^HR, hazard ratio; ^b^CI, confidence interval; ^*^ *p* <0.05.

### MUC4/Y stable overexpression

To investigate the role of MUC4/Y in pancreatic cancer, PANC-1 cells, which do not express endogenous MUC4 [22], were infected with viral supernatant containing either MUC4/Y or empty lentiviral expression plasmids for effective MUC4/Y overexpression or to serve as controls, respectively. Pooled populations of Puro-tagged stable transfected sub-lines were selected using 10% DMEM containing puromycin (2.0 μg/mL).

We examined stable MUC4/Y overexpression in PANC-1-MUC4/Y cells at transcript and protein level using real-time PCR and immunoblotting, respectively; the negative and blank controls were mock-transfected cells (PANC-1-EV) and parental wild-type PANC-1 cells, respectively.

**Figure 3:**
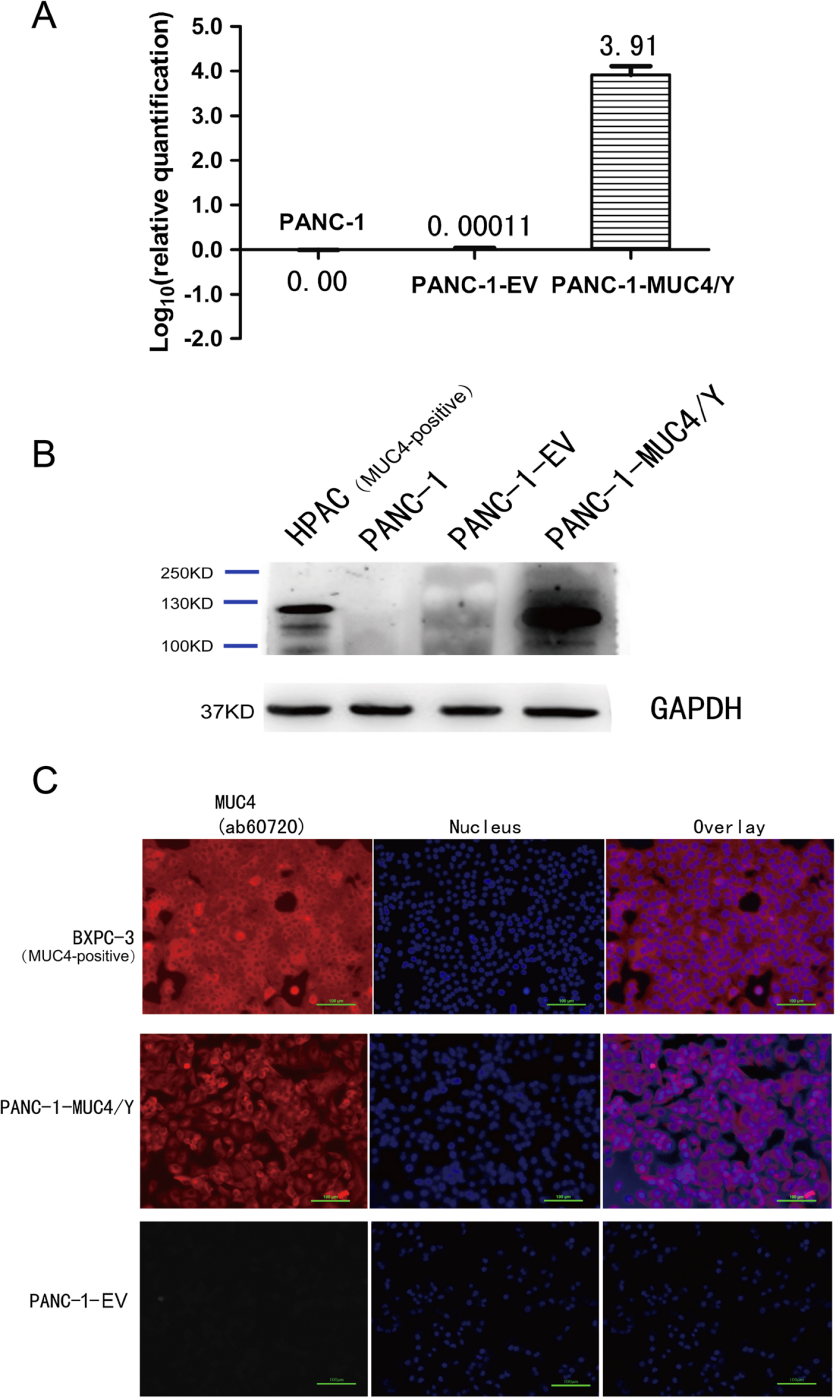
**MUC4/Y expression and subcellular localization in PANC-1 cells. (A)** Real-time PCR using specific primers and TaqMan probe to examine MUC4/Y transcript expression in PANC-1-EV cells and PANC-1-MUC4/Y cells. The level of target gene expression in the PANC-1-MUC4/Y cells was 9912-fold and 9808-fold higher than that of the blank control and negative control, respectively. **(B)** Western blot confirmation of MUC4/Y protein expression. Total protein from cell extracts was resolved on precast gels. The signal was detected using an electrochemiluminescence reagent kit. **(C)** Immunofluorescence demonstrating MUC4/Y subcellular localization similar to that of wild-type MUC4. The pancreatic cancer cell lines of HPAC and BXPC-3 is MUC4 positive-expression as positive control for specific antibody.

We assessed MUC4/Y quantitatively using real-time RT-PCR with gene-specific priming (Figure 1A). The level of *MUC4/Y* mRNA expression was normalized to that of 18S rRNA. MUC4/Y expression in PANC-1-MUC4/Y cells was 9912-fold and 9808-fold higher than that in the blank and negative controls, respectively (Figure 3A).

In agreement with the mRNA findings, western blotting confirmed MUC4/Y overexpression in the PANC-1-MUC4/Y cells. The selected monoclonal antibody (#ab60720; Abcam) is specifically directed against amino acids 79–189 of human MUC4, which are included in the protein expressed by the *MUC4/Y* target gene. Figure 3B depicts a band that had migrated a distance consistent with the expected 133-kDa molecular weight of MUC4/Y. The blank and negative control cells were negative for MUC4 expression. HPAC, a MUC4-overexpressing pancreatic cell line, was used as a positive control for specific antibody [13].

We used the MUC4 monoclonal antibody to examine the subcellular localization of MUC4/Y using immunofluorescence. The pancreatic cancer cell line BXPC-3 is MUC4 positive-expression as positive control [13]. In BXPC-3 cells, there was both membranous and cytoplasmic staining for MUC4 (Figure 3C). A similar distribution was observed for PANC-1-MUC4/Y cells. MUC4/Y and wild-type MUC4 had similar sub-cellular localization, indicating similar MUC4 and MUC4/Y processing in the cells.

### MUC4/Y contributed to enhance proliferation under low-nutritional-pressure, anti-apoptosis, motility, invasiveness, and HUVEC endotube formation in PANC-1 cells in vitro

**Figure 4:**
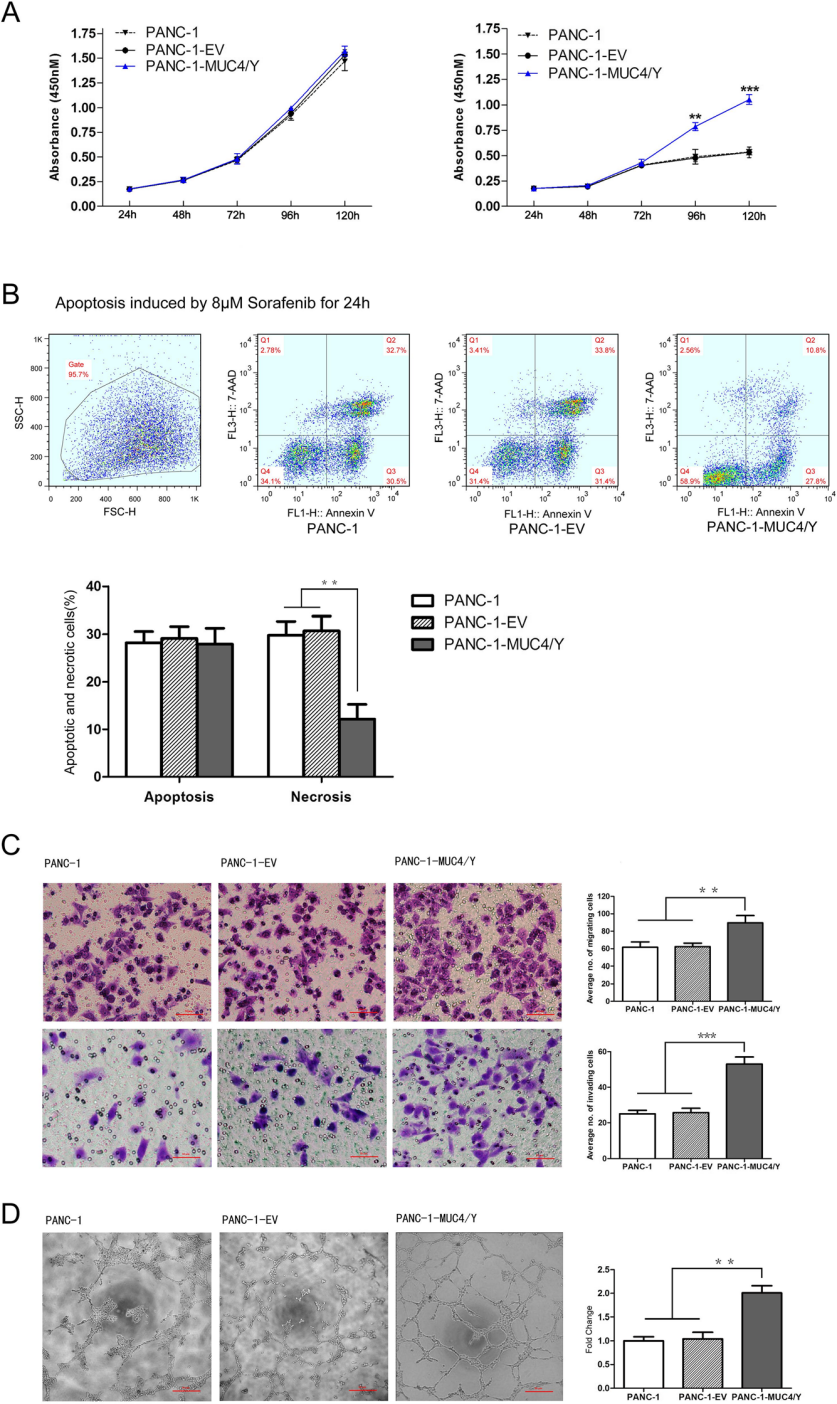
**MUC4/Y enhances PANC-1 cell malignant activity** ***in vitro*** **.** Data from three repeated experiments are presented as means ± SD. **P <0.01, ***P <0.001 vs. controls. **(A)** MUC4/Y enhanced *in vitro* proliferation of PANC-1 cells under stress from low nutritional status. The absorbance values of cells at different time points were detected with WST-8 dye. Cells were maintained in medium containing 10% serum (left) and 1% serum (right). **(B)** MUC4/Y increased resistance to apoptotic reagents, i.e., sorafenib. Representative templates of FACS analysis showing the proportion of cells positive for annexin V and 7-AAD (top right quadrant) representing the percentage of necrotic cells; the proportion of cells that were annexin V–positive and 7-AAD–negative (bottom right quadrant) represented the percentage of apoptotic cells (top). Bar denotes the percentage of apoptotic and necrotic cells in PANC-1–derived clones (bottom). **(C)** MUC4/Y affected pancreatic cancer cell metastatic potential *in vitro*. Bar graph shows the number of PANC-1–derived clones that had migrated or invaded through the Matrigel. **(D)** MUC4/Y enhanced cancer cell–associated HUVEC endotube formation. Bar denotes the fold increase of the number of endotubes compared to the blank control.

Figure 4A shows that there was no significant difference for PANC-1-MUC4/Y cells grown in 10% serum compared to the controls. However, under stress from low nutritional status (1% serum), there were significant increases in the proliferation of PANC-1-MUC4/Y cells compared to the blank controls at 96 h and 120 h (*P* = 0.003, 0.000, respectively), suggesting that MUC4/Y enhances *in vitro* proliferation of PANC-1 cells under stress from low nutritional status.

To examine whether MUC4/Y enhances resistance to apoptosis in pancreatic cancer cells, PANC-1-MUC4/Y cells and the controls were treated with 8 μM sorafenib for 24 h and apoptosis was assessed using flow cytometry. The proportion of cells positive for annexin V and 7-AAD was significantly decreased (*P* <0.01, respectively) compared to the blank and negative controls (Figure 4B), suggesting that MUC4/Y plays an anti-apoptotic role in pancreatic cancer *in vitro*.

We used Transwells without or with Matrigel-coated membranes to examine cell migration and invasion, respectively, *in vitro*. The average number of migrating cells indicated the significantly increased migration ability of PANC-1-MUC4/Y cells compared to that of the controls (*P* <0.01, respectively). Figure 4C shows that the number of PANC-1-MUC4/Y cells invading through the Matrigel was significantly higher than that of the controls (*P* <0.001, respectively). These data suggest that MUC4/Y can affect the metastatic potential of pancreatic cancer cells *in vitro*.

We also investigated whether MUC4/Y modulates the ability of PANC-1 cells to influence endotube formation by vascular endothelial cells. We used a 2-chamber co-culture system to demonstrate the interaction between PANC-1 cells and HUVECs. Co-culturing HUVECs with PANC-1-MUC4/Y cells significantly enhanced HUVEC endotube formation (*P* <0.01) compared to co-culture with the negative and blank controls (Figure 4D).

### MUC4/Y contributed to increase tumor growth and metastasis with rising proliferative activity, MVD, and metastasis incidence and decreased apoptosis in vivo

To analyze the role of MUC4/Y *in vivo*, we developed subcutaneous and orthotopic models using PANC-1–derived clones.

**Figure 5:**
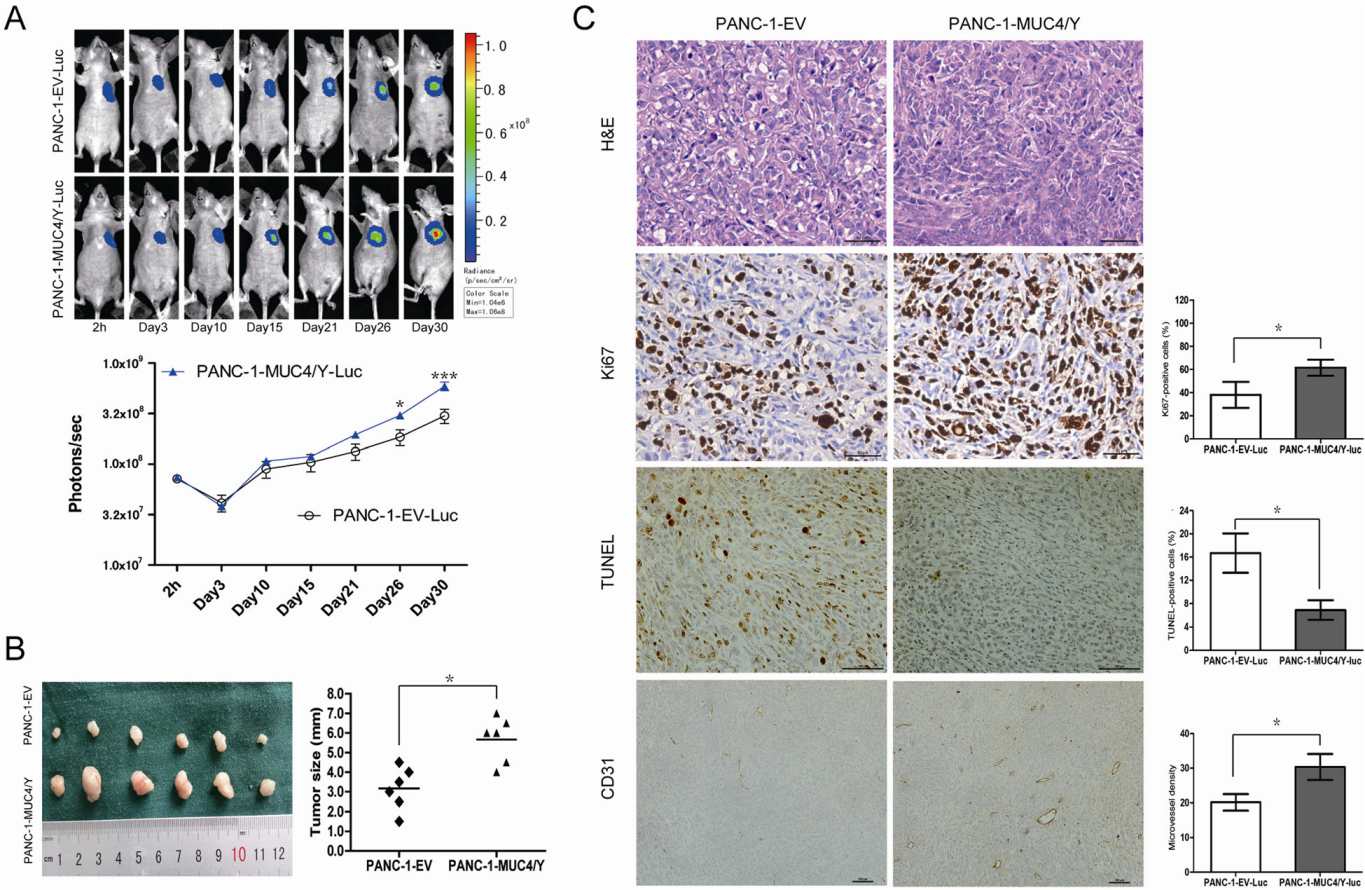
**MUC4/Y contributed to increase tumor growth with rising proliferative activity and MVD and decreased apoptosis in vivo. (A)** *In vivo* BLI showing the tumor growth rates over time in the subcutaneous model. Up: Representative luminescence images for each group. Down: Tumor growth rates indicated by bioluminescence (photons/s) in PANC-1-MUC4/Y-Luc (*n* = 6) and control (*n* = 6) BALB/c nude mice at 2 hours and 3, 10, 15, 21, 26, 30 days after tumor cell injection; bars, SE. *P <0.05, ***P <0.001. **(B)** Subcutaneous tumors and their size (mm) from two groups measured at the 30-day time point when mice were sacrificed. Scatter dot, tumor size of every mouse; the center horizontal line, mean. *P <0.05. **(C)** Histological and IHC analysis of subcutaneous tumors. Histologically, there was no difference between subcutaneous tumors of the PANC-1-MUC4/Y-Luc group and control groups (H&E staining, ×400 magnification). Ki67, TUNEL, and CD31 staining is of paraffin-embedded sections from solid tumors. Micrographs are representative images of two groups (Ki67, TUNEL, CD31: ×400, 200, 100 magnification, respectively). Charts depict the mean proportion of Ki67- and TUNEL-positive cells and the average number of CD31-positive microvessels per field, respectively. Five fields per slide and at least five slides per group were examined and compared using the Student *t*-test. Columns, mean; bars, SD; *P <0.05.

In the subcutaneous model, we used PANC-1-MUC4/Y-Luc cells (negative control: PANC-1-EV-Luc) to obtain *in vivo* BLI, and the mean tumor growth rates at different time points determined by BLI (photons/s) were compared using repeated-measures analysis of variance (ANOVA) to identify subject-by-time profiles. Subcutaneously localized luciferase activity was identical in the two groups at the 2-hour time point, but at the 26, 30-day time point, there was a statistically significant increase in the bioluminescent signal in PANC-1-MUC4/Y-Luc cells compared to the negative control (Figure 5A), indicating that at the later stages, significant difference of tumor growth arised due to MUC4/Y-overexpression *in vivo*.

Moreover, at the 30-day time point, mice were sacrificed, and tumor sizes were measured. There was significant difference in tumor size between the two groups (Figure 5B, *P* <0.05).

Ki-67 is an excellent marker of cell proliferation. Histologic evaluation of the tumors confirmed that MUC4/Y-overexpression markedly increased the fraction of Ki-67-positive tumor cells (the Ki-67 labelling index) compared to the controls (*P* <0.05, Figure 5C, the Second Line).

TUNEL was performed to assess the number of apoptotic cells *in vivo*. The proportion of TUNEL-positive PANC-1-MUC4/Y-Luc cells was significantly lower compared to the controls (*P* <0.05, Figure 5C, the Third Line).

We also assessed the effects of MUC4/Y overexpression on tumor vasculature by MVD analysis via CD31 staining of tumors vasculature (Figure 5C, the Fourth Line). Compared to the controls, MUC4/Y significantly increased MVD in the subcutaneous tumors (*P* <0.05).

**Figure 6:**
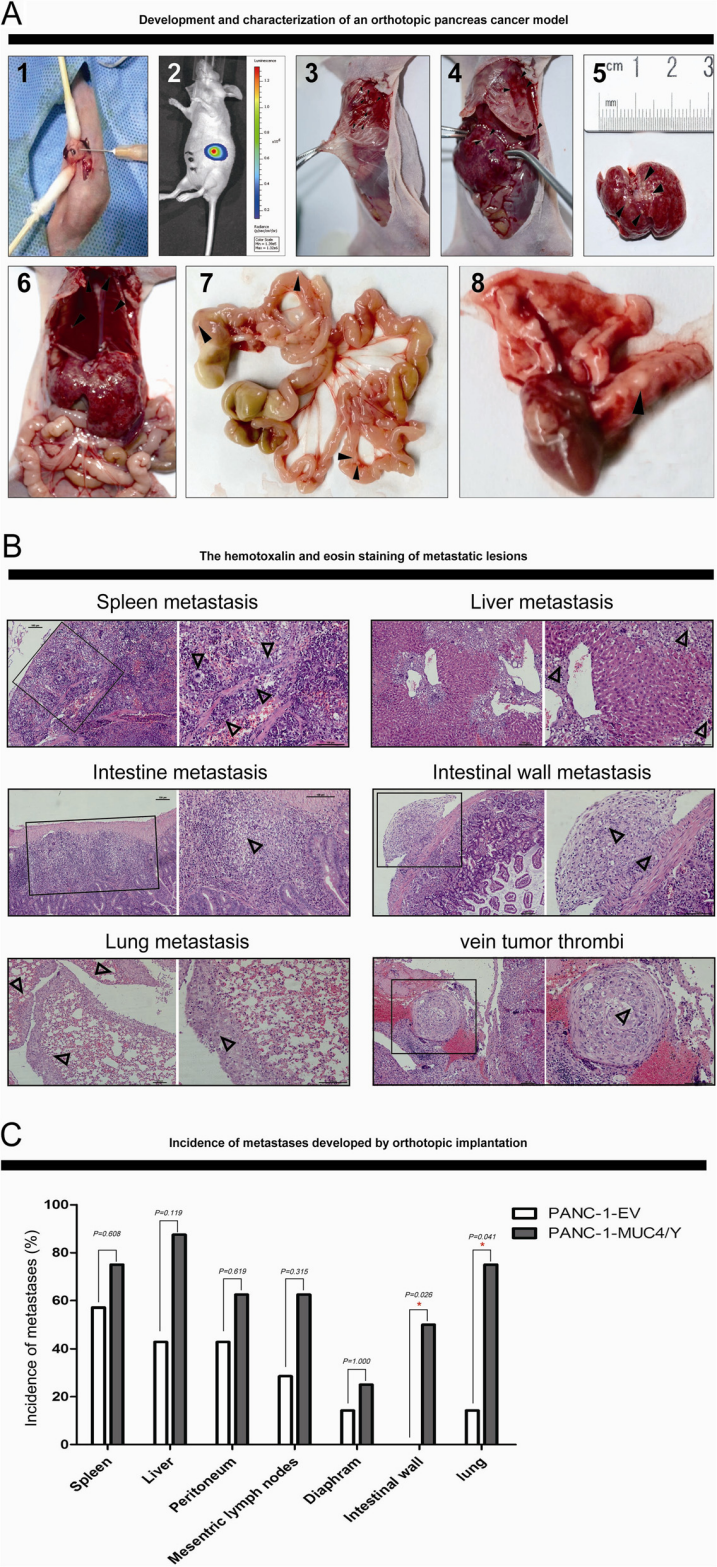
**Development of orthotopic pancreatic cancer model for observation of metastasis. (A)** Development of orthotopic pancreatic cancer and gross inspection. 1. PANC-1–derived clones were orthotopically implanted in the parenchyma of the pancreatic head. 2. Luciferase activity imaging at two days following tumor cell injection confirming orthotopic implantation at the injection site without cell spillage. 3–8. Metastasis to the peritoneum, peritoneum and liver, liver, diaphragm and liver, mesenteric lymph node and intestinal wall, and lung, indicated by arrowheads (▲), were obvious macroscopically. (**B)** Histological analysis of metastatic lesions. Metastatic lesions of the spleen, liver, downward mucosa of the intestine, intestinal wall, lung, vein tumor thrombi (H&E staining, left: ×100 magnification; right: ×200 magnification, Δ indicates tumor). **(C)** Increased incidences of metastasis at different sites in MUC4/Y overexpression group compared to the control group (*n* = 8 per group). Comparisons between groups were tested with the chi-square test, *P <0.05.

Given the importance of the microenvironment in cancer pathogenesis, we developed a pancreatic orthotopic model to observe metastasis using PANC-1–derived clones with or without MUC4/Y overexpression. We confirmed metastasis by visual and histological inspection (Figure 6A-B). We also used *in vivo* BLI to confirm that the metastases were not related to tumor cell spillage at the time of orthotopic implantation (Figure 6A-2). The overall tumor incidence between the two groups was not significantly different. However, there were higher incidences of metastasis at different sites in the MUC4/Y-overexpressing group compared to the control groups (Figure 6C), including tumor spread in the spleen (6/8 vs. 4/7, *P* = 0.608), liver (7/8 vs. 3/7, *P* = 0.119), peritoneum (5/8 vs. 3/7, *P* = 0.619), mesenteric lymph nodes (5/8 vs. 2/7, *P* = 0.315), diaphragm (2/8 vs. 1/7, *P* = 1.000), intestinal wall (4/8 vs. 0/7, *P* = 0.026), and lung (6/8 vs. 1/7, *P* = 0.041), suggesting that MUC4/Y overexpression promoted tumor metastasis (intestinal wall) and distant metastasis (lung) significantly effectively. Vein tumor thrombi were obvious in the PANC-1-MUC4/Y group (Figure 6B-6), suggesting that MUC4/Y overexpression aids in PANC-1 cell hematogenous metastasis.

In sum, the above-mentioned data confirmed that the overexpression-MUC4-Y resulted in increasing tumor growth and metastasis in vivo.

### DEG screening and functional annotation for global mRNA analysis of PANC-1-MUC4/Y- cells

Using DGE analysis, we compared PANC-1-MUC4/Y cells to the blank and negative controls to identify the overlapping differential genes based on re-sampling tests and elimination of negative vector and random insert effects. Comparative analyses of MUC4/Y-associated signatures revealed 3446 and 4149 differential genes for the blank and negative control cell lines, respectively, including 1575 overlapping genes [see Additional file 1: Table S4 lists the DEGs, intersection set of PANC-1-MUC4/Y compared to the controls, respectively, absolute value of log2 ratio ≥1].

To understand the DEG global functions, we carried out enrichment analysis of GO function [23] and the KEGG pathway [24]. Additional file 1: Table S5 lists the detailed results of the GO enrichment analysis, indicating that MUC4/Y overexpression not only resulted in transcriptional change of the transmembrane, which is intrinsic to membrane and extracellular region–associated proteins for cytokine activities that control tissue and cell survival, growth, differentiation, and effector function, but also altered interactions between the cell and its surroundings, and intracellular signaling and signal transmission.

Enrichment analysis with KEGG confirmed that the set of 1575 DEGs was enriched for pathways not only correlated to cancer, but also were associated with MUC4, as shown in the literature database (Additional file 1: Table S6), and was consistent with the GO functional enrichment analysis.

### Verification of DEGs related with the malignant functions of MUC4/Y and VEGF family molecules

**Figure 7:**
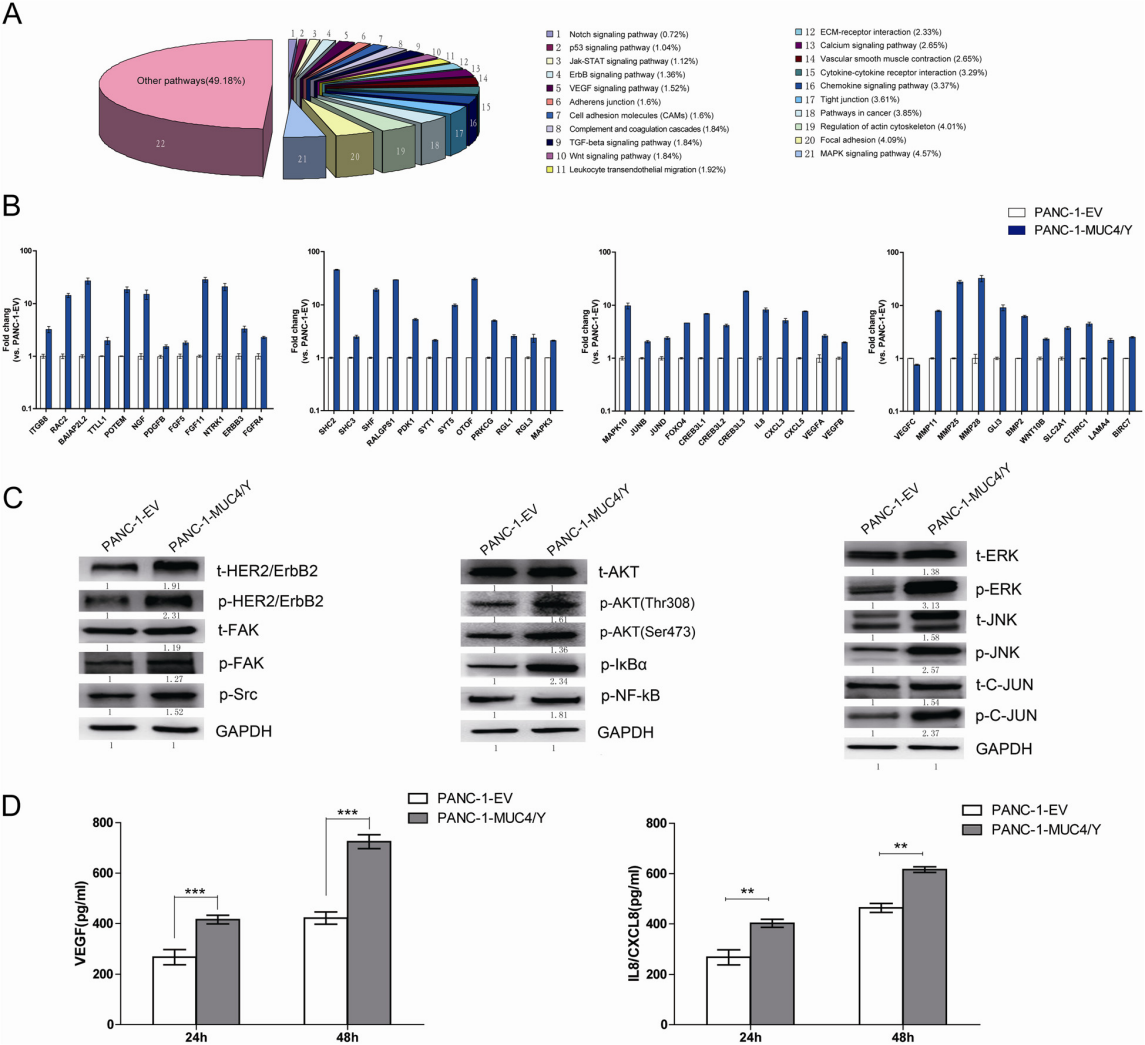
**qRT-PCR, western blotting, and ELISA verification of DEGs related to the malignant functions of MUC4/Y and VEGF family molecules. (A)** Distribution of DEGs in the main pathways related to oncogenic transformation showing the change derived from MUC4/Y overexpression–associated molecular signatures; the proportions of each pathway (represented by different colors) are graphed. The DEG list for annotations is the intersection set of PANC-1-MUC4/Y compared to the controls; absolute value of log2 ratio ≥1. **(B)** Representative qPCR validation results of 44 DEGs and VEGF family molecules (VEGFA, B, C) in PANC-1-MUC4/Y and control cell. **(C)** Western blot analysis verified a significant upregulation of t-HER2/ErbB2, p-HER2/ErbB2, t-FAK, p-FAK, p-Src, p-AKT(Thr308), p-AKT(Ser473), p-IκBα, p-NF-kB, t-ERK, p-ERK, t-JNK, p-JNK, and p-C-JUN in PANC-1-MUC4/Y compared with control. **(D)** ELISA of VEGF and IL8/CXCL8 using cell culture supernatants. Left: VEGF; right: IL8/CXCL8 production from PANC-1–derived clones incubated for 24 h or 48 h. Bars denote mean ± SD, ***P <0.001, **P <0.01.

To evaluate the malignant functions of MUC4/Y, we summarized the distribution of DEGs in a number of pathways markedly related to the malignance of cancer using Database for Annotation, Visualization, and Integrated Discovery (DAVID, LIB) software and KEGG annotations (Figure 7A).

For qPCR validation, we selected a batch of DEG molecules that play roles in the main pathways related to oncogenic transformation, based on the literature database. The independent qRT-PCR results for these genes were consistent with the DGE results (Figure 7B), indicating that our sequencing approach and analytical pipeline were reliable.

For western blotting, we selected a batch of proteins related to DEG molecules, especially protein phosphorylation (Figure 7C). There was a MUC4/Y-dependent increase of ErbB2 phosphorylation activation, paralleled by the upregulation of key molecules (Ras, Src, focal adhesion kinase [FAK], extracellular signal–regulated kinase [ERK], c-Jun amino-terminal kinase [JNK], AKT, nuclear factor κB [NFκB], inhibitor of NFκB [IκBα], c-Jun), as either total or phosphorylated proteins, in the downstream signaling pathways.

Based on the results demonstrating the effect of MUC4/Y associated with angiogenesis and metastasis in malignant progression of pancreatic cancer, we verified the expression change of the key factors in cell culture supernatants with ELISA, combined with validation of their mRNA expression by qRT-PCR. MUC4/Y overexpression up-regulated the transcriptional level of VEGFA and VEGFB compared to the control, but down-regulated VEGFC transcriptional level (Figure 7B). MUC4/Y overexpression significantly increased PANC-1-MUC4/Y cell output of both VEGF (main detection of VEGFA) and IL8/CXCL8 into the medium after 24-h or 48-h incubation compared to the control (Figure 7D).

## Discussion

We designed specific primers and a TaqMan probe (Figure 1A) for MUC4/Y and verified their specificity by sequencing the PCR products. We applied a strict statistical test which found that with the rise in levels of TNM stage, the level of MUC4/Y mRNA expression increased significantly. MUC4/Y expression at TNM stage IA + IB was significantly lower than that at TNM stage IIA, IIB-2 or III + IV, and its expression at IIA, IIB (including IIB-1,2) was significantly lower than that at III + IV, respectively. These suggest that MUC4/Y relates to the invasion, progression and distant metastases, which also coincides with the results of in vitro and in vivo experiments based on stable MUC4/Y-overexpressing pancreatic cancer cell models. The single discrepancy that we observed in the staging system is relatively minor. There is no significant difference between MUC4/Y expression at TNM stage IA + IB and IIB-1(regional nodal involvement), and there was also no significant difference between MUC4/Y expression at TNM stage IIA and IIB-2 (regional nodal involvement). This may be because of the level of MUC4/Y mRNA expression is not associated with regional lymph node metastasis. The results of positive correlation between MUC4/Y and MUC4 expression levels in PDAC clinical samples also suggest that MUC4/Y might play similar roles as MUC4 in the malignant progression of PDAC patient.

Every univariate factor-specific overall survival was estimated by using the Kaplan-Meier method and compared with log-rank tests. Our results showed those variables identified by the Kaplan-Meier method as having predictive value were the same ones as by univariate analysis (Figure 2 and Table 2), which would be included in the multivariate analysis. Then the effect of potential confounding variables on survival was examined by using the multivariate Cox proportional hazard model after adjusting for confounding, including global and covariate-specific tests. Our results showed that as same as tumor location, tumor differentiation, TNM staging, serum level of CA19-9, serum level of CA50, serum level of CEA and MUC4 mRNA expression status, MUC4/Y mRNA expression level was also significant predictor of survival in univariate analysis. But only tumor location, tumor differentiation, TNM stage, serum CA19-9, and MUC4 expression were independently associated with survival differences in a multivariate Cox proportional hazards model, which are in accordance with previous reports [10,11,25,26]. These results suggest that the effect of MUC4/Y expression is less than the effect of MUC4 expression on survival. That might be because *MUC4/Y* is only the one of splice variants of MUC4.

Using lentiviral transfection, we constructed and identified pancreatic cancer PANC-1 cell lines that stably overexpressed the MUC4/Y gene. The subcellular localization is similar to that of wild-type MUC4, indicating that all functional domains are expressed and processed accurately in our MUC4/Y-overexpressing cell model in agreement with that of wild-type MUC4. Based on the cell models, our results reveal that MUC4/Y contributes to enhance malignant activities that were observed in the experimental assay in vitro and in vivo, including proliferation, evasion or resistance to apoptosis, angiogenesis and metastasis.

Meanwhile, We noted that MUC4/Y enhanced in vitro proliferation of PANC-1 cells under stress from low nutritional status. Coincidentally, bioluminescence imaging revealed that MUC4/Y contributed to enhance the tumor growth rates in the subcutaneous model at the later stages (day26, 30 after injection). We speculate that MUC4/Y may be initiate survival signals, increase energy-generating ability or prevent the apoptosis to resist the stress from the lack of growth factors and energies. Additionally, the differences of growth rates between MUC4/Y-overexpressed PANC-1 cells and control in earlier stage were not observed probably due to the tumors not growing large enough to undergo an angiogenic switch. Furthermore, the detailed analyses of transcriptome changes caused by MUC4/Y gene support this inference. MUC4/Y not only up-regulates a series of genes (GLI family zinc finger 3, *GLI3* [27]; bone morphogenetic protein 2, *BMP2* [28]; wingless-type MMTV integration site family member 10B, *WNT10B* [29], *IL8* [30], *NGF* [31]) related to the enhancement of cancer cell proliferation and increased transcription of apoptotic protein inhibitors (baculoviral IAP repeat containing 7, *BIRC7* [32];), but also up-regulates glucose transporter (*SLC2A1/GLUT1*) related to the increased energy-generating ability.

We developed a pancreatic orthotopic model to observe tumor spontaneous metastases by visual and histological inspection. Tumors developed at organ sites similarly as metastatic colonization by human PDAC metastases, including direct invasion to adjacent organs (spleen, intestine, peritoneum), regional lymph node metastases or dissemination by those (mesenteric lymph nodes, diaphragm), and distant metastasis (liver, lung). Our data indicate that MUC4/Y overexpression promoted adjacent metastasis (intestinal wall) and distant metastasis (lung) significantly effectively, which also is consistent with the positive correlation of MUC4/Y mRNA expression with tumor invasion and distant metastases in human PDAC clinic samples. Additionally, the effects of MUC4/Y on ascending malignant abilities(proliferation, anti-apoptosis, motility, invasiveness, angiogenesis) of tumor cells can also affect the metastatic potential of pancreatic cancer cells during multi-steps of metastasis process, including invasion, intravasation, extravasation, metastatic colonization. Notably, our data provide evidence for MUC4/Y upregulates ITGB8 downstream of the actin cytoskeleton pathway (ITGB8-FAK-Cas/CrkII/DOCK180 complex-RAC- IRSp53- WAVE2-Arp2/3-F-Actin/PFN complex) to affect actin dynamics and cancer cell motility [33].

Angiogenesis is critical for the continuous growth of tumors and the development of metastases. Our study provides evidence that MUC4/Y plays an initial role in tumor angiogenesis. We find and verify that MUC4/Y overexpression contributed to an array of crucial factors involved in angiogenesis: IL8 [34], CXCL3, CXCL5 [35], matrix metalloproteinase (MMP)11 [36], MMP25 [37], MMP28 [38], VEGF [39]. Interestingly, MUC4/Y up-regulates the transcriptional level of VEGFA and VEGFB, but down-regulates VEGFC transcriptional level compared to the control. VEGF represents a family with multiple functions that affect tumor growth and metastasis. Specifically, VEGF-A is crucial for tumor angiogenesis and plays a key role in endothelial cell growth, migrationl, and permeability as a ligand for VEGFR-1 and VEGFR-2. VEGFB regulates the formation of blood vessels and are involved in endothelial cell physiology as a ligand for VEGFR-1 and NRP-1(neuropilin-1). The up-regulation of both of the above explains MUC4/Y contributes to tumor angiogenesis. The other isoform of VEGF, VEGFC and its specific receptor VEGFR-3 compose an essential signal pathway for lymphatic vessel growth in physiological and pathological conditions, more importantly in lymphatic spread of metastases. Intriguingly, the down-regulation of VEGFC coincides with MUC4/Y has no effect on regional lymph node metastases in pancreatic orthotopic model- mice and PDAC clinic samples, although more experiments may need to be confirmed.

**Figure 8:**
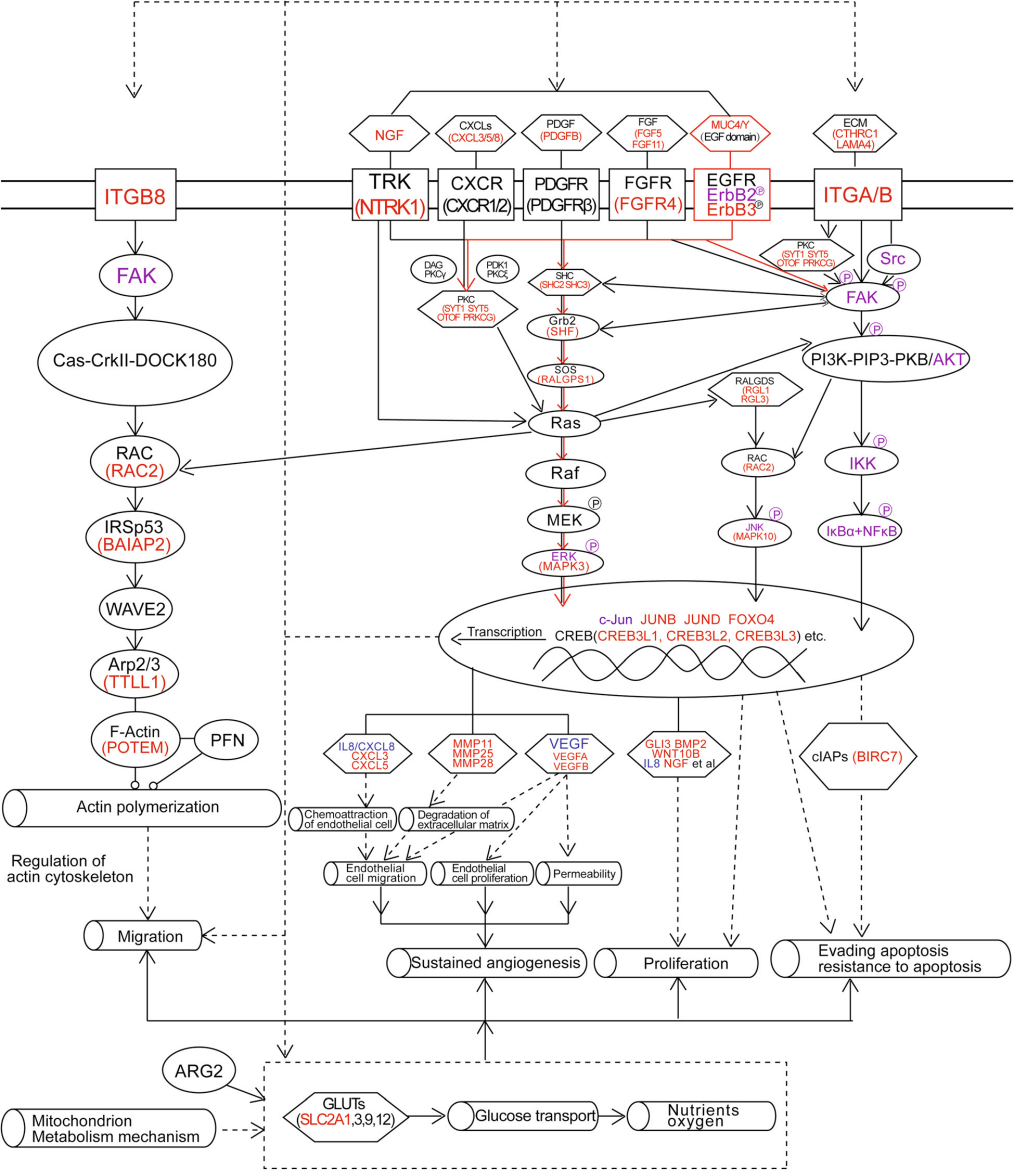
**MUC4/Y-dependent pathways involved in malignant activity.** Scheme depicts gene complexes and families (hexagons), membrane receptors (rectangles), function (hollow cylinders), and others (ovals). Red, purple, and blue key factors in the signaling pathways were verified by qRT-PCR, western blotting, and ELISA, respectively. Red lines denote the triggers.

Significantly, as shown in Figure 8, we investigated transcriptome changes caused by MUC4/Y expression using DGE technology, bioinformatics analysis and experimental verification, and have depicted a condensed version focusing on the malignant activity related pathways.

Importantly, we have found that EGF domain in MUC4/Y and its interaction with ErbB2 and ErbB3 receptors trigger intrinsic protein–tyrosine kinase activity and further activate intracellular signaling pathways (e.g., mitogen-activated protein kinase [MAPK], phosphatidylinositol-3-kinase [PI3K]–Akt, protein kinase C [PKC] pathways), and coactivate transcription of the downstream effector molecules to mediate malignant functions of tumor cell, specifically, the production or upregulation of cytokines, growth factors, extracellular matrix (ECM), integrins (ITGs), and corresponding membrane receptors (CXCL3/5/8, nerve growth factor [NGF], neurotrophic tyrosine receptor kinase type 1 [NTRK1], platelet-derived growth factor beta polypeptide [PDGFB], fibroblast growth factor [FGF]5/11, FGFR4, collagen triple helix repeat-containing 1 [CTHRC1], laminin alpha 4 [LAMA4], ITGB8; range: 1.27 ± 0.20-fold to 27.92 ± 3.58-fold increase). Notably, the transcription of cytokines, growth factors, ECM, and ITGs, and the interaction between them and the corresponding membrane receptors can in turn lead to upregulation of the signal transduction cascades, including the MAPK, PI3K–Akt, and PKC pathways. Thus, MUC4/Y expression that concomitantly elicits transcript-level upregulation of an array of molecules creates the positive feedback regulatory loops that would result in sustained upregulation of oncogenetic and progression signaling and lead to complex interplay or crosstalk between several signaling pathways.

Significantly, the above analysis and the novel finding that MUC4/Y triggers positive feedback loops related to malignant activity not only suggest that tumor cells can engage MUC4/Y to express survival factors concomitantly to resist adverse conditions in the microenvironment, e.g., in the circulatory systems during metastasis, but also suggest that tumor cells can exploit MUC4/Y expression to affect the tumor milieu by increasing the expression of cytokines, growth factors, and adhesion molecules, although the lack of the TR domain mainly relates to cell–ECM and cell–cell interplay [40,41].

Taken together, we provide evidence that supports the effects of MUC4/Y in the malignant progression of pancreatic cancer. These results on functional studies are consistent with similar trends compared to the results reported from experimental model of over-expression of rat orthologue of MUC4 (rMuc4/SMC) [42,43] and mini-MUC4[21], or silencing/knockdown of MUC4 (sh-MUC4) [40,44,45]. Together with the positive correlation of MUC4/Y with MUC4 expression in PDAC clinical samples, we have validated our reasoning that MUC4/Y is involved in malignant progression similarly to FL-MUC4 and, consequently, MUC4/Y and its domain-lacking models may be valuable tools for the further dissection of MUC4-mediated functions and mechanisms.

Although we have pointed out the over-expression and alternative splicing of MUC4 may create a favorable environment for tumor progression by triggering malignancy-related positive feedback loops, it would be more expected to further investigate the interaction and network between those key mediators and their receptors, between involved signal pathways, as well as between transcription factors in the formation of circuit using new approaches like systems biology and clinical bioinformatics.

## Conclusions

The huge length of its gene fragment restricts the functional and mechanism research of human MUC4. As one of its splice variants, MUC4/Y with coding sequence is mostly as similar as FL-MUC4. The level of MUC4/Y mRNA expression in PDAC clinical samples indicates that MUC4/Y is significantly positive-correlated with tumor invasion and distant metastases. The positive correlation between MUC4/Y and MUC4 expression levels in PDAC clinical samples also suggests that MUC4/Y might play similar roles as MUC4 in the malignant progression of pancreatic cancer. Forced-expression MUC4/Y has sub-cellular localization similar to that of wild-type MUC4, suggesting similar protein processing in the transfection cell model. Functional studies show that MUC4/Y enhances cell model malignant activity *in vitro and in vivo*, including proliferation under low-nutritional-pressure, resistance to apoptosis, motility, invasiveness, angiogenesis, and distant metastasis. Mechanism studies indicate the novel finding that MUC4/Y triggers malignancy-related positive feedback loops for concomitantly up-regulating the expression of survival factors to resist adverse microenvironment and increasing the expression of an array of cytokines and adhesion molecules to affect the tumor milieu. In light of the enormity of the potential regulatory circuitry in cancer afforded by MUC4 and/or MUC4/Y, repressing MUC4 transcription, inhibiting post-transcriptional regulation, including alternative splicing, or blocking various pathways simultaneously may be helpful for controlling malignant progression. MUC4/Y- expression model is proven to valuable tool for the further dissection of MUC4-mediated functions and mechanisms.

## Additional file

Additional file 1: Table S1. AJCC 6th Edition TNM Staging System for Pancreatic Cancer. **Table S2.** Specific primer sequences used in qRT-PCR assays for the validation of DGE results. **Table S3.** The list of the specific antibodies and concentrations used in the Western blot assays for the validation of DGE results. **Table S4.** List of 1575 differentially expressed genes (DEGs), and the intersection set of PANC-1-MUC4/Y compared to two controls, respectively; absolute value of log2 ratio ≥1. (Status:↑, upregulation of gene expression levels in MUC4/Y compared to controls; ↓, downregulation of gene expression levels in MUC4/Y compared to controls). **Table S5.** Functional categories of common differentially expressed genes (DEGs) in PANC-1 cells of overexpressing MUC4/Y as compared with both control cell lines. **Table S6.** Representative KEGG pathways from signaling pathway impact analysis of DEGs in PANC-1 cells of over-expressing MUC4/Y compared with both control cell lines. DEGs were annotated with the indicated KEGG database [7,46–58].

## Abbreviations

7-AAD: 7-amino-actinomycin D
ANOVA: Analysis of variance
BLI: Bioluminescence imaging
BIRC7: Baculoviral IAP repeat containing 7
BMP2: Bone morphogenetic protein 2
cDNA: Complementary DNA
CI: Confidence interval
Ct: Threshold cycle value
CTHRC1: Collagen triple helix repeat-containing 1
DEGs: Differentially expressed genes
DGE: Digital gene expression
DMEM: Dulbecco’s modified Eagle’s medium
ECM: Extracellular matrix
EGF: Epidermal growth factor-like domain
ELISA: Enzyme-linked immunosorbent assay
ERK: Extracellular signal–regulated kinase
EV: Empty lentiviral vectors
FACS: Fluorescence-activated cell sorter
FAK: Focal adhesion kinase
FBS: Fetal calf serum
FGF: Fibroblast growth factor
FL-MUC4: Full-length MUC4
GAPDH: Glyceraldehyde-3-phosphate dehydrogenase
GFP: Green fluorescent protein
GLI3: GLI family zinc finger 3
GLUT: Glucose transporter
GO: Gene Ontology
H&E: Hematoxylin and eosin
HER2: Activation of human EGF receptor type 2
HR: Hazard ratio
HUVECs: Human umbilical vein endothelial cells
IL8: Interleukin-8
IHC: Immunohistochemistry
IκBα: Inhibitor of NFκB
ITGs: Integrins
JNK: c-Jun amino-terminal kinase
KEGG: Kyoto Encyclopedia of Genes and Genomes
LAMA4: Laminin alpha 4
MAPK: Mitogen-activated protein kinase
MVD: Microvessel density
NGF: Nerve growth factor
NFκB: Nuclear factor κB
NIDO: The nidogen (NIDO) -like domain
NTRK1: Neurotrophic tyrosine receptor kinase type 1
OS: Overall survival
PBS: Phosphate-buffered saline
PDAC: Pancreatic ductal adenocarcinoma
PDGFB: Platelet-derived growth factor beta polypeptide
PDK: Pyruvate dehydrogenase kinase
PI3K: Phosphatidylinositol-3-kinase
PKC: Protein kinase C
qRT-PCR: Quantitative reverse transcription polymerase chain reaction
ROC: Receiver operating characteristic
RT: Reverse transcription
SD: Standard deviation
SV: Splice variants
TPM: Transcripts per million
TNM: Tumor-node-metastasis
TR: Tandem repeat
TUNEL: Terminal deoxynucleotidyl transferase–mediated dUTP nick end labeling
WNT10B: Wingless-type MMTV integration site family member 10B
VEGF: Vascular endothelial growth factor
