# Supplementary material for: Specific-detection of clinical samples, systematic functional investigations, and transcriptome analysis reveals that splice variant MUC4/Y contributes to the malignant progression of pancreatic cancer by triggering malignancy-related positive feedback loops signaling
Source: J Transl Med. 2014 Nov 4;12:309. doi: 10.1186/s12967-014-0309-8 (PMC4236435; doi:10.1186/s12967-014-0309-8)
Supplement: Additional file 1: Table S1. — AJCC 6th Edition TNM Staging System for Pancreatic Cancer. Table S2. Specific primer sequences used in qRT-PCR assays for the validation of DGE results. Table S3. The list of the specific antibodies and concentrations used in the Western blot assays for the validation of DGE results. Table S4. List of 1575 differentially expressed genes (DEGs), and the intersection set of PANC-1-MUC4/Y compared to two controls, respectively; absolute value of log2 ratio ≥1. (Status:↑, upregulation of gene expression levels in MUC4/Y compared to controls; ↓, downregulation of gene expression levels in MUC4/Y compared to controls). Table S5. Functional categories of common differentially expressed genes (DEGs) in PANC-1 cells of overexpressing MUC4/Y as compared with both control cell lines. Table S6. Representative KEGG pathways from signaling pathway impact analysis of DEGs in PANC-1 cells of over-expressing MUC4/Y compared with both control cell lines. DEGs were annotated with the indicated KEGG database [7,46–58]. [file 12967_2014_309_MOESM1_ESM.doc]

**Table S1. AJCC 6th Edition TNM Staging System for Pancreatic Cancer.**

| **Definitions of TNM** | | | | |
| --- | --- | --- | --- | --- |
| TX | Primary tumor cannot be assessed | | | |
| T0 | No evidence of primary tumory | | | |
| Tis | Carcinoma in situ | | | |
| T1 | Tumor limited to the pancreas, 2 cm or less in greatest diameter | | | |
| T2 | Tumor limited to the pancreas, greater than 2 cm in greatest diameter | | | |
| **T3*** | Tumor extends beyond pancreas but no involvement of celiac axis or superior mestenteric artery | | | |
| T4 | Tumor involves the celiac axis or the superior mestenteric artery (unresectable) | | | |
| NX | Regional nodes cannot be assessed | | | |
| N0 | No regional lymph node metastasis | | | |
| N1 | Regional lymph node metastasis | | | |
| MX | Distant metastasis cannot be assessed | | | |
| M0 | No distantant metastasis | | | |
| M1 | Distant metastasis | | | |
| Stage grouping | | | | |
| Stage 0 | Tis | N0 | M0 | Localized within pancreas |
| Stage IA | T1 | N0 | M0 | Localized within pancreas |
| Stage IB | T2 | N0 | M0 | Localized within pancreas |
| Stage IIA | T3 | N0 | M0 | Locally invasive, resectable |
| **Stage IIB*** | T1 | N1 | M0 | Locally invasive, resectable |
|  | T2 | N1 | M0 | Locally invasive, resectable |
|  | T3 | N1 | M0 | Locally invasive, resectable |
| Stage III | T4 | Any N | M0 | Locally advanced, unresectable |
| Stage IV | Any T | Any N | M1 | Distant metastases |
| ***Here Stage IIB can be divided in two groups: one defined as IIB-1, i.e. Tumor limited to the pancreas, Regional lymph node metastasis, No distantant metastasis (T1+T2, N1, M0); the other defined as IIB-2, i.e. Tumor extends beyond pancreas but locally invasive, Regional lymph node metastasis, No distantant metastasis (T3, N1, M0).** | | | | |

**Table S2. Specific primer sequences used in qRT-PCR assays for the validation of DGE results.**

| Symbol | Gene ID | Forward Prime | Reverse Primer |
| --- | --- | --- | --- |
| **ITGB8** | 3696 | ACCAGGAGAAGTGTCTATCCAG | CCAAGACGAAAGTCACGGGA |
| **RAC2** | 5880 | CAACGCCTTTCCCGGAGAG | TCCGTCTGTGGATAGGAGAGC |
| **BAIAP2** | 80115 | GCCGAGGTCTACTTCAGTGC | GCTGGGTGTCAGACATCTGC |
| **TTLL1** | 25809 | GTGACAGAAAACGAGGACTGG | CAGCTTCAACGCTGAACACA |
| **POTEM** | 641455 | ACCAAACTTCGCAGAAAGCC | TCGCCTTCACCATTCCAGTT |
| **NGF** | 4803 | GGCAGACCCGCAACATTACT | CACCACCGACCTCGAAGTC |
| **PDGFB** | 5155 | CTCGATCCGCTCCTTTGATGA | CGTTGGTGCGGTCTATGAG |
| **FGF5** | 2250 | CACTGATAGGAACCCTAGAGGC | CAGATGGAAACCGATGCCC |
| **FGF11** | 2256 | TGAGGGACTGCTCTACAGTTC | GGCGTACAGGACGTAGTAATTC |
| **NTRK1** | 4914 | AACCTCACCATCGTGAAGAGT | TGAAGGAGAGATTCAGGCGAC |
| **ERBB3** | 2065 | GACCCAGGTCTACGATGGGAA | GTGAGCTGAGTCAAGCGGAG |
| **FGFR4** | 2264 | GAGGGGCCGCCTAGAGATT | CAGGACGATCATGGAGCCT |
| **SHC2** | 25759 | TCCTACGTCGTGCGGTACAT | CCTCATGGAGCCGGTTGATG |
| **SHC3** | 53358 | CTTGGGGTGCATTGAAGTTCT | GGCTTCCCTGGTAATTTGTGTT |
| **SHF** | 90525 | CCGTGGCTAATGGGCCAAA | CAAACGGGTCCGCATAGTCTT |
| **RALGPS1** | 9649 | CAAGAGCTATGATGCCGTTGT | GGCAAGACTGTGTTTCTCCTTC |
| **PDK1** | 5163 | CTGTGATACGGATCAGAAACCG | TCCACCAAACAATAAAGAGTGCT |
| **SYT1** | 6857 | GTGAGCGAGAGTCACCATGAG | CCCACGGTGGCAATGGAAT |
| **SYT5** | 6861 | AGACGCTGAACCCTCACTTTG | CGAAGTCGTACACCGCCAT |
| **OTOF** | 9381 | CAACAAGCGTGTCGCCTATG | TCCTTGCGCTGTTTGCTGA |
| **PRKCG** | 5582 | GAAGACCCGAACGGTGAAAG | CTCCGAGACGCCAAAGGAC |
| **RGL1** | 23179 | GCAAGATGGCTAGGGGTTGAA | TGCCAGCTTTTATGGTCCTGA |
| **RGL3** | 57139 | CTGGGGTGAAGAGACCGAG | TGCTGGTTCGATAGTGGAGGA |
| **MAPK3** | 5595 | CTACACGCAGTTGCAGTACAT | CAGCAGGATCTGGATCTCCC |
| **MAPK10** | 5602 | CAGATGGAATTAGACCATGAGCG | TCAATGTGCAATCAGACTTGACT |
| **JUNB** | 3726 | ACGACTCATACACAGCTACGG | GCTCGGTTTCAGGAGTTTGTAGT |
| **JUND** | 3727 | TCATCATCCAGTCCAACGGG | TTCTGCTTGTGTAAATCCTCCAG |
| **FOXO4** | 4303 | GGCTGCCGCGATCATAGAC | GGCTGGTTAGCGATCTCTGG |
| **CREB3L1** | 90993 | CCTCCCGAAGCCTCCTATTCT | GGGGTTGATTTCCCAGCCA |
| **CREB3L2** | 64764 | CAGAGAAGAGTGTGTCAATGGAG | CTGGTGGTAATGTGGGTGAAG |
| **CREB3L3** | 84699 | GCCCTGCCTCTCCTATCATC | ACGGTGAGATTGCATCGTGG |
| **IL8** | 3576 | ACTGAGAGTGATTGAGAGTGGAC | AACCCTCTGCACCCAGTTTTC |
| **CXCL3** | 2921 | CGCCCAAACCGAAGTCATAG | GCTCCCCTTGTTCAGTATCTTTT |
| **CXCL5** | 6374 | AGCTGCGTTGCGTTTGTTTAC | TGGCGAACACTTGCAGATTAC |
| **VEGFA** | 7422 | AGGGCAGAATCATCACGAAGT | AGGGTCTCGATTGGATGGCA |
| **VEGFB** | 7423 | GAGATGTCCCTGGAAGAACACA | GAGTGGGATGGGTGATGTCAG |
| **VEGFC** | 7424 | GAGGAGCAGTTACGGTCTGTG | TCCTTTCCTTAGCTGACACTTGT |
| **MMP11** | 4320 | CCGCAACCGACAGAAGAGG | ATCGCTCCATACCTTTAGGGC |
| **MMP25** | 64386 | GACTGGCTGACTCGCTATGG | TGATGGCATCGCGCAACTT |
| **MMP28** | 79148 | TACCAACAGTTATGCGGCCTG | CAAAGCGTTTCTTACGCCTCA |
| **GLI3** | 2737 | GAAGTGCTCCACTCGAACAGA | GTGGCTGCATAGTGATTGCG |
| **BMP2** | 650 | ACCCGCTGTCTTCTAGCGT | TTTCAGGCCGAACATGCTGAG |
| **WNT10B** | 7480 | GTGAGCGAGACCCCACTATG | CACTCTGTAACCTTGCACTCATC |
| **SLC2A1** | 6513 | GGCCAAGAGTGTGCTAAAGAA | ACAGCGTTGATGCCAGACAG |
| **CTHRC1** | 115908 | CAATGGCATTCCGGGTACAC | GTACACTCCGCAATTTTCCCAA |
| **LAMA4** | 3910 | CCACACTCGTCCTTCTCTCTC | AGTTTCCGAACTGACCTAGCC |
| **BIRC7** | 79444 | GCTCTGAGGAGTTGCGTCTG | CACACTGTGGACAAAGTCTCTT |

**Table S3. The list of the specific antibodies and concentrations used in the Western blot assays for the validation of DGE results.**

| Antibody | Brand | Article Number | Molecular Wt. | Isotype | Dilution |
| --- | --- | --- | --- | --- | --- |
| HER2/ErbB2 | CST(USA) | #2242 | 185KDa | Rabbit | 1:1000 |
| Phospho-HER2 (Tyrosine 1248) | CST(USA) | #2247 | 185KDa | Rabbit | 1:1000 |
| Phospho-Src (Tyr 416) | CST(USA) | #6943 | 60KDa | Rabbit | 1:1000 |
| FAK | CST(USA) | #3285 | 125KDa | Rabbit | 1:1000 |
| Phospho-FAK (Tyr 397) | CST(USA) | #3283 | 125KDa | Rabbit | 1:1000 |
| AKT(pan) (C67E7) | CST(USA) | #4691 | 60KDa | Rabbit | 1:1000 |
| Phospho-AKT(Thr308) | CST(USA) | #2965 | 60KDa | Rabbit | 1:1000 |
| Phospho-AKT(Ser473) | CST(USA) | #3787 | 60KDa | Rabbit | 1:1000 |
| Phospho-IκBα(Ser32) | CST(USA) | #2859 | 40KDa | Rabbit | 1:1000 |
| Phospho-NF-kBp65(Ser536) | CST(USA) | #3033 | 65KDa | Rabbit | 1:1000 |
| Ras | CST(USA) | #8955 | 21KDa | Rabbit | 1:1000 |
| Erk1/2 | CST(USA) | #4695 | 42,44KDa | Rabbit | 1:1000 |
| Phospho-Erk1/2 (Thr202/Tyr204) | CST(USA) | #4370 | 42,44KDa | Rabbit | 1:2000 |
| SAPK/JNK (56G8) | CST(USA) | #9258 | 46,54KDa | Rabbit | 1:1000 |
| Phospho-SAPK/JNK (Thr183/Tyr185) | CST(USA) | #4668 | 46,54KDa | Rabbit | 1:1000 |
| c-Jun (60A8) | CST(USA) | #9165 | 43,48KDa | Rabbit | 1:1000 |
| Phospho-c-Jun (Ser63) | CST(USA) | #2361 | 48KDa | Rabbit | 1:1000 |
| GAPDH | Beyotime(China) | #AG019 | 36KDa | Mouse | 1:1000 |
| Anti-rabbit IgG^HRP^ | CST(USA) | #7074 |  |  | 1:1000 |
| Anti-mouse IgG ^HRP^ | CST(USA) | #7076 |  |  | 1:1000 |

**Table S4. List of 1575 differentially expressed genes (DEGs), and the intersection set of PANC-1-MUC4/Y compared to two controls, respectively; absolute value of log2 ratio ≥1. (Status:↑, upregulation of gene expression levels in MUC4/Y compared to controls; ↓, downregulation of gene expression levels in MUC4/Y compared to controls).**

| Symbol | Gene ID | status | Symbol | Gene ID | status | Symbol | Gene ID | status | Symbol | Gene ID | status |
| --- | --- | --- | --- | --- | --- | --- | --- | --- | --- | --- | --- |
| TLE2 | 7089 | ↑ | SLC23A1 | 9963 | ↑ | DOC2A | 8448 | ↑ | REEP2 | 51308 | ↑ |
| CREB3L1 | 90993 | ↑ | SELE | 6401 | ↑ | KDR | 3791 | ↑ | HSD17B14 | 51171 | ↑ |
| TTYH2 | 94015 | ↑ | USP49 | 25862 | ↑ | GPC2 | 221914 | ↑ | MT1A | 4489 | ↑ |
| TMC8 | 147138 | ↑ | LOC147670 | 147670 | ↑ | TP53I11 | 9537 | ↑ | ITGA10 | 8515 | ↑ |
| LRP1 | 4035 | ↑ | MIR4284 | 1E+08 | ↑ | LMF1 | 64788 | ↑ | HES2 | 54626 | ↑ |
| EFNA3 | 1944 | ↑ | TF | 7018 | ↑ | SCD | 6319 | ↑ | KCNN4 | 3783 | ↑ |
| FRAT1 | 10023 | ↑ | EFHB | 151651 | ↑ | RASA4 | 10156 | ↑ | BRWD3 | 254065 | ↑ |
| CORO2A | 7464 | ↑ | LOC100288123 | 1E+08 | ↑ | C5 | 727 | ↑ | RNF208 | 727800 | ↑ |
| C7orf10 | 79783 | ↑ | CECR2 | 27443 | ↑ | TTC39B | 158219 | ↑ | DLX4 | 1748 | ↑ |
| NXPH4 | 11247 | ↑ | NGF | 4803 | ↑ | TMOD2 | 29767 | ↑ | NLRX1 | 79671 | ↑ |
| IL8 | 3576 | ↑ | UGT8 | 7368 | ↑ | CAPN8 | 388743 | ↑ | TMEM200B | 399474 | ↑ |
| UPK1A | 11045 | ↑ | JAZF1-AS1 | 1E+08 | ↑ | EBI3 | 10148 | ↑ | TMEM151A | 256472 | ↑ |
| TUBB4A | 10382 | ↑ | PLAGL1 | 5325 | ↑ | PTPRB | 5787 | ↑ | RBL2 | 5934 | ↑ |
| MYO15B | 80022 | ↑ | TRIM78P | 117852 | ↑ | C16orf55 | 124045 | ↑ | SLC16A3 | 9123 | ↑ |
| SPTSSB | 165679 | ↑ | SEL1L3 | 23231 | ↑ | PTPRG | 5793 | ↑ | OSCAR | 126014 | ↑ |
| CCDC114 | 93233 | ↑ | LOC100507050 | 1.01E+08 | ↑ | CRABP2 | 1382 | ↑ | MEG3 | 55384 | ↑ |
| DEPTOR | 64798 | ↑ | ZNF221 | 7638 | ↑ | ENO2 | 2026 | ↑ | LOC100133091 | 1E+08 | ↑ |
| CYP2U1 | 113612 | ↑ | C2orf84 | 653140 | ↑ | C9orf37 | 85026 | ↑ | LOC100128071 | 1E+08 | ↑ |
| ESPN | 83715 | ↑ | PYGM | 5837 | ↑ | SOBP | 55084 | ↑ | ECHDC3 | 79746 | ↑ |
| ARHGEF4 | 50649 | ↑ | ANKRD45 | 339416 | ↑ | TBC1D8B | 54885 | ↑ | KLHL28 | 54813 | ↑ |
| SHC2 | 25759 | ↑ | CXCR2P1 | 3580 | ↑ | FAM114A1 | 92689 | ↑ | CLDN4 | 1364 | ↑ |
| LTBR | 4055 | ↑ | MGAT3 | 4248 | ↑ | ST6GALNAC2 | 10610 | ↑ | ST5 | 6764 | ↑ |
| GNG7 | 2788 | ↑ | CTNNA3 | 29119 | ↑ | FAM131A | 131408 | ↑ | METRNL | 284207 | ↑ |
| KLHL13 | 90293 | ↑ | FGB | 2244 | ↑ | ACVR2B | 93 | ↑ | CTSF | 8722 | ↑ |
| PPP1R1B | 84152 | ↑ | TGM4 | 7047 | ↑ | SLC44A2 | 57153 | ↑ | IQSEC2 | 23096 | ↑ |
| C11orf45 | 219833 | ↑ | LINC00478 | 388815 | ↑ | RPS6KA2 | 6196 | ↑ | TMEM120A | 83862 | ↑ |
| SULT2B1 | 6820 | ↑ | KIAA1958 | 158405 | ↑ | STON1 | 11037 | ↑ | IFIT2 | 3433 | ↑ |
| FAM211B | 388886 | ↑ | SLC7A2 | 6542 | ↑ | ACAD10 | 80724 | ↑ | ECH1 | 1891 | ↑ |
| C6orf132 | 647024 | ↑ | BTBD11 | 121551 | ↑ | PLXNB1 | 5364 | ↑ | MAP3K6 | 9064 | ↑ |
| NOTCH3 | 4854 | ↑ | PDE4C | 5143 | ↑ | P4HA1 | 5033 | ↑ | AIG1 | 51390 | ↑ |
| RHBDL2 | 54933 | ↑ | RUNX1-IT1 | 80215 | ↑ | SELENBP1 | 8991 | ↑ | VWA1 | 64856 | ↑ |
| RAB17 | 64284 | ↑ | PACSIN1 | 29993 | ↑ | DHRS3 | 9249 | ↑ | BARX1 | 56033 | ↑ |
| PLCD4 | 84812 | ↑ | DHRS4 | 10901 | ↑ | LOC644172 | 644172 | ↑ | MOV10 | 4343 | ↑ |
| ZNF540 | 163255 | ↑ | AURKAPS1 | 6791 | ↑ | VASH1 | 22846 | ↑ | WDR54 | 84058 | ↑ |
| LINC00174 | 285908 | ↑ | PTGFRN | 5738 | ↑ | EFR3B | 22979 | ↑ | EHBP1L1 | 254102 | ↑ |
| SYT17 | 51760 | ↑ | SLC10A5 | 347051 | ↑ | ZNF345 | 25850 | ↑ | WASF3 | 10810 | ↑ |
| ASPHD2 | 57168 | ↑ | CHRNA4 | 1137 | ↑ | LOC284751 | 284751 | ↑ | C20orf96 | 140680 | ↑ |
| SMAD9 | 4093 | ↑ | FLJ39639 | 283876 | ↑ | SAA2 | 6289 | ↑ | DSTNP2 | 171220 | ↑ |
| NPHS1 | 4868 | ↑ | LRRC39 | 127495 | ↑ | GCNT1 | 2650 | ↑ | GPX4 | 2879 | ↑ |
| PRPH | 5630 | ↑ | TEX11 | 56159 | ↑ | KIAA1274 | 27143 | ↑ | PDGFB | 5155 | ↑ |
| GABRD | 2563 | ↑ | EXOC6B | 23233 | ↑ | FAM116B | 414918 | ↑ | SLC4A5 | 57835 | ↑ |
| ZNF41 | 7592 | ↑ | GFRA3 | 2676 | ↑ | RHPN1 | 114822 | ↑ | CCNG1 | 900 | ↑ |
| CES1 | 1066 | ↑ | MGAT4A | 11320 | ↑ | KLHL24 | 54800 | ↑ | ADCY9 | 115 | ↑ |
| DNHD1 | 144132 | ↑ | TLL2 | 7093 | ↑ | RGL1 | 23179 | ↑ | MAP2 | 4133 | ↑ |
| SLC16A14 | 151473 | ↑ | SLC15A3 | 51296 | ↑ | NLGN2 | 57555 | ↑ | ZNF418 | 147686 | ↑ |
| AATK | 9625 | ↑ | SLC12A5 | 57468 | ↑ | PRSS16 | 10279 | ↑ | NTF4 | 4909 | ↑ |
| DNAJC12 | 56521 | ↑ | TMEM88B | 643965 | ↑ | FGF5 | 2250 | ↑ | WNT10B | 7480 | ↑ |
| LINC00173 | 1E+08 | ↑ | CLCA2 | 9635 | ↑ | PRELID2 | 153768 | ↑ | FDPSL2A | 619190 | ↑ |
| KCNH3 | 23416 | ↑ | APOL1 | 8542 | ↑ | H1FX-AS1 | 339942 | ↑ | GDNF | 2668 | ↑ |
| DICER1-AS1 | 400242 | ↑ | LOC285033 | 285033 | ↑ | ADAMTS2 | 9509 | ↑ | GNB3 | 2784 | ↑ |
| FMN1 | 342184 | ↑ | CLMP | 79827 | ↑ | KIRREL2 | 84063 | ↑ | LOC100272228 | 1E+08 | ↑ |
| SLC29A4 | 222962 | ↑ | FAM78A | 286336 | ↑ | COL9A3 | 1299 | ↑ | DIO3OS | 64150 | ↑ |
| CCL20 | 6364 | ↑ | CCDC149 | 91050 | ↑ | VILL | 50853 | ↑ | FNDC4 | 64838 | ↑ |
| ZNF862 | 643641 | ↑ | ZNF763 | 284390 | ↑ | ARRB1 | 408 | ↑ | TSTD1 | 1E+08 | ↑ |
| CLSTN3 | 9746 | ↑ | VWCE | 220001 | ↑ | PTPRCAP | 5790 | ↑ | SLC2A3 | 6515 | ↑ |
| LOC653160 | 653160 | ↑ | ZNF443 | 10224 | ↑ | SLC5A5 | 6528 | ↑ | PNRC1 | 10957 | ↑ |
| BTN3A3 | 10384 | ↑ | GABRB1 | 2560 | ↑ | SEC31B | 25956 | ↑ | TECR | 9524 | ↑ |
| PSD | 5662 | ↑ | C22orf23 | 84645 | ↑ | C16orf79 | 283870 | ↑ | PDE9A | 5152 | ↑ |
| PPFIA4 | 8497 | ↑ | SYN2 | 6854 | ↑ | BTN3A2 | 11118 | ↑ | INHA | 3623 | ↑ |
| CADM4 | 199731 | ↑ | GBP6 | 163351 | ↑ | TFF3 | 7033 | ↑ | WFIKKN1 | 117166 | ↑ |
| TNFSF15 | 9966 | ↑ | HOGA1 | 112817 | ↑ | FAM83E | 54854 | ↑ | GLIS1 | 148979 | ↑ |
| FAM171A2 | 284069 | ↑ | MRVI1-AS1 | 1E+08 | ↑ | ARHGEF10L | 55160 | ↑ | LOC100130275 | 1E+08 | ↑ |
| MMP28 | 79148 | ↑ | ERVW-1 | 30816 | ↑ | PCED1B | 91523 | ↑ | ZNF500 | 26048 | ↑ |
| PNCK | 139728 | ↑ | WNK2 | 65268 | ↑ | KAZALD1 | 81621 | ↑ | ZNF853 | 54753 | ↑ |
| IFI27 | 3429 | ↑ | CFB | 629 | ↑ | LINC00511 | 400619 | ↑ | LOC100499227 | 1E+08 | ↑ |
| HOXA4 | 3201 | ↑ | UBE2Q2P2 | 1E+08 | ↑ | SRRM3 | 222183 | ↑ | CRIP2 | 1397 | ↑ |
| BEND2 | 139105 | ↑ | RGS7 | 6000 | ↑ | CORO2B | 10391 | ↑ | ZNF485 | 220992 | ↑ |
| PLAC1 | 10761 | ↑ | RGS9 | 8787 | ↑ | NEURL2 | 140825 | ↑ | CNTNAP1 | 8506 | ↑ |
| C1RL | 51279 | ↑ | BMP8B | 656 | ↑ | CD14 | 929 | ↑ | ZNF385A | 25946 | ↑ |
| CERS4 | 79603 | ↑ | PARD3B | 117583 | ↑ | ITGB8 | 3696 | ↑ | KIAA1161 | 57462 | ↑ |
| LOC100129518 | 1E+08 | ↑ | ATP1B2 | 482 | ↑ | CHST13 | 166012 | ↑ | KLHDC8B | 200942 | ↑ |
| NEUROD1 | 4760 | ↑ | VIL1 | 7429 | ↑ | FAM155B | 27112 | ↑ | HCST | 10870 | ↑ |
| SYT8 | 90019 | ↑ | BEND7 | 222389 | ↑ | LOC100289230 | 1E+08 | ↑ | C10orf47 | 254427 | ↑ |
| CPAMD8 | 27151 | ↑ | ALDH3A1 | 218 | ↑ | DBP | 1628 | ↑ | EXOC4 | 60412 | ↑ |
| DAND5 | 199699 | ↑ | CACNA1A | 773 | ↑ | KIF7 | 374654 | ↑ | GK | 2710 | ↑ |
| ZNF497 | 162968 | ↑ | HIST1H3B | 8358 | ↑ | LYNX1 | 66004 | ↑ | IFT172 | 26160 | ↑ |
| CLIC3 | 9022 | ↑ | CCBP2 | 1238 | ↑ | AMOT | 154796 | ↑ | PC | 5091 | ↑ |
| RASGRP2 | 10235 | ↑ | HIST2H2AC | 8338 | ↑ | CLDN3 | 1365 | ↑ | TLE4 | 7091 | ↑ |
| SLC7A7 | 9056 | ↑ | EFCAB5 | 374786 | ↑ | TLCD2 | 727910 | ↑ | ZNF395 | 55893 | ↑ |
| PLCD1 | 5333 | ↑ | MAPK13 | 5603 | ↑ | PLIN2 | 123 | ↑ | CACNA1H | 8912 | ↑ |
| KLHL3 | 26249 | ↑ | PCDH15 | 65217 | ↑ | FSD1 | 79187 | ↑ | TNFRSF18 | 8784 | ↑ |
| C1orf38 | 9473 | ↑ | LOC100506012 | 1.01E+08 | ↑ | TM6SF2 | 53345 | ↑ | PYCARD | 29108 | ↓ |
| COL9A2 | 1298 | ↑ | FAM92A1P2 | 403315 | ↑ | TNFRSF25 | 8718 | ↑ | NXPH2 | 11249 | ↓ |
| LCN2 | 3934 | ↑ | LOC100302650 | 1E+08 | ↑ | WDR66 | 144406 | ↑ | CRYBB2 | 1415 | ↓ |
| PTGIS | 5740 | ↑ | LOC389332 | 389332 | ↑ | C1QTNF3 | 114899 | ↑ | ZNF708 | 7562 | ↓ |
| RAB11FIP4 | 84440 | ↑ | RELN | 5649 | ↑ | ASS1 | 445 | ↑ | CTU2 | 348180 | ↓ |
| RSPH9 | 221421 | ↑ | GALNT14 | 79623 | ↑ | SPIRE2 | 84501 | ↑ | GJB3 | 2707 | ↓ |
| NMUR1 | 10316 | ↑ | GRIN3A | 116443 | ↑ | REEP6 | 92840 | ↑ | HEATR3 | 55027 | ↓ |
| TCAM1P | 146771 | ↑ | ZNF160 | 90338 | ↑ | STX1B | 112755 | ↑ | ADRB2 | 154 | ↓ |
| HAS2-AS1 | 594842 | ↑ | TIE1 | 7075 | ↑ | SLC4A3 | 6508 | ↑ | EFTUD1 | 79631 | ↓ |
| LOC100506046 | 1.01E+08 | ↑ | KCNN2 | 3781 | ↑ | MAGEH1 | 28986 | ↑ | THBD | 7056 | ↓ |
| HSPA12A | 259217 | ↑ | EMILIN3 | 90187 | ↑ | CACFD1 | 11094 | ↑ | NKX6-2 | 84504 | ↓ |
| KREMEN1 | 83999 | ↑ | SCGB1B2P | 643719 | ↑ | GPSM1 | 26086 | ↑ | AMHR2 | 269 | ↓ |
| LOC388152 | 388152 | ↑ | CALHM3 | 119395 | ↑ | FBXL20 | 84961 | ↑ | FLJ35024 | 401491 | ↓ |
| ACPL2 | 92370 | ↑ | PTPLAD2 | 401494 | ↑ | ROBO3 | 64221 | ↑ | MYO18A | 399687 | ↓ |
| NRTN | 4902 | ↑ | IL20RB | 53833 | ↑ | CBX7 | 23492 | ↑ | LOC439994 | 439994 | ↓ |
| ACSS3 | 79611 | ↑ | MSI1 | 4440 | ↑ | HDAC11 | 79885 | ↑ | PTPLB | 201562 | ↓ |
| NCKAP5 | 344148 | ↑ | LOC150197 | 150197 | ↑ | IDUA | 3425 | ↑ | NCR3LG1 | 374383 | ↓ |
| ADARB2 | 105 | ↑ | BMP8A | 353500 | ↑ | RALGPS1 | 9649 | ↑ | KCNRG | 283518 | ↓ |
| HLA-DMA | 3108 | ↑ | EPHX2 | 2053 | ↑ | C10orf10 | 11067 | ↑ | FSIP1 | 161835 | ↓ |
| GDPD1 | 284161 | ↑ | DEGS2 | 123099 | ↑ | TMEM173 | 340061 | ↑ | P4HA3 | 283208 | ↓ |
| PLEKHB1 | 58473 | ↑ | DEFB132 | 400830 | ↑ | PRRX2 | 51450 | ↑ | FBXO4 | 26272 | ↓ |
| GPIHBP1 | 338328 | ↑ | OR51B5 | 282763 | ↑ | ARVCF | 421 | ↑ | MUC16 | 94025 | ↓ |
| PCSK4 | 54760 | ↑ | FLJ22763 | 401081 | ↑ | PPP2R3A | 5523 | ↑ | MRC1 | 4360 | ↓ |
| BCAM | 4059 | ↑ | MRGPRF | 116535 | ↑ | FAM57B | 83723 | ↑ | BICD1 | 636 | ↓ |
| C1QTNF8 | 390664 | ↑ | DHX58 | 79132 | ↑ | RAP1GAP2 | 23108 | ↑ | ALG11 | 440138 | ↓ |
| CEACAM1 | 634 | ↑ | GLB1L | 79411 | ↑ | TMEM63A | 9725 | ↑ | OGDHL | 55753 | ↓ |
| PTPRU | 10076 | ↑ | UBE2Q2P3 | 1E+08 | ↑ | HDHD1 | 8226 | ↑ | HIST1H2BK | 85236 | ↓ |
| NXNL2 | 158046 | ↑ | C9orf172 | 389813 | ↑ | RCN3 | 57333 | ↑ | WBP11 | 51729 | ↓ |
| CCT6P3 | 643180 | ↑ | INPP5J | 27124 | ↑ | CELSR2 | 1952 | ↑ | CFC1B | 653275 | ↓ |
| DEF6 | 50619 | ↑ | WAS | 7454 | ↑ | THBS3 | 7059 | ↑ | PLCB4 | 5332 | ↓ |
| CPT1C | 126129 | ↑ | MGC3771 | 81854 | ↑ | SLC9A5 | 6553 | ↑ | CCL14 | 6358 | ↓ |
| SPTBN2 | 6712 | ↑ | FAM167B | 84734 | ↑ | TMEM91 | 641649 | ↑ | CACNA2D1 | 781 | ↓ |
| LOC400643 | 400643 | ↑ | CNR2 | 1269 | ↑ | QPRT | 23475 | ↑ | PPTC7 | 160760 | ↓ |
| C9orf9 | 11092 | ↑ | CCL28 | 56477 | ↑ | CTSK | 1513 | ↑ | FAM86EP | 348926 | ↓ |
| SLC25A27 | 9481 | ↑ | HPGD | 3248 | ↑ | SCARB1 | 949 | ↑ | ISL1 | 3670 | ↓ |
| MIR1273D | 1E+08 | ↑ | SNORD45C | 692085 | ↑ | ASMTL | 8623 | ↑ | DUSP2 | 1844 | ↓ |
| RAB40B | 10966 | ↑ | CXCL5 | 6374 | ↑ | ASIC3 | 9311 | ↑ | DQX1 | 165545 | ↓ |
| CA9 | 768 | ↑ | AGMO | 392636 | ↑ | CDA | 978 | ↑ | MCM3AP-AS1 | 114044 | ↓ |
| LOC100134868 | 1E+08 | ↑ | LAMB4 | 22798 | ↑ | DUSP9 | 1852 | ↑ | CFL1P1 | 142913 | ↓ |
| PCDH18 | 54510 | ↑ | SPEF1 | 25876 | ↑ | PINK1 | 65018 | ↑ | MCC | 4163 | ↓ |
| GSDMB | 55876 | ↑ | MYH11 | 4629 | ↑ | ATP2B4 | 493 | ↑ | CDK8 | 1024 | ↓ |
| RPA4 | 29935 | ↑ | LOX | 4015 | ↑ | WISP2 | 8839 | ↑ | CEMP1 | 752014 | ↓ |
| BACH2 | 60468 | ↑ | MPZ | 4359 | ↑ | SMARCD3 | 6604 | ↑ | SNORA1 | 677792 | ↓ |
| KLHL29 | 114818 | ↑ | BMP2 | 650 | ↑ | DNM1 | 1759 | ↑ | KIF26A | 26153 | ↓ |
| VWDE | 221806 | ↑ | TMEM92 | 162461 | ↑ | RNF128 | 79589 | ↑ | SCML2 | 10389 | ↓ |
| UPK2 | 7379 | ↑ | DPY19L2P1 | 554236 | ↑ | LOC100527964 | 1.01E+08 | ↑ | RAB36 | 9609 | ↓ |
| ALDH1L2 | 160428 | ↑ | CIB3 | 117286 | ↑ | IL17RD | 54756 | ↑ | D4S234E | 27065 | ↓ |
| C1orf115 | 79762 | ↑ | BTG2 | 7832 | ↑ | INTU | 27152 | ↑ | DKFZP686I15217 | 401232 | ↓ |
| LOC440356 | 440356 | ↑ | IQGAP2 | 10788 | ↑ | TP73 | 7161 | ↑ | NRL | 4901 | ↓ |
| SHF | 90525 | ↑ | PRICKLE2 | 166336 | ↑ | CNN1 | 1264 | ↑ | ZRANB3 | 84083 | ↓ |
| HBA1 | 3039 | ↑ | CRB3 | 92359 | ↑ | LOC100272217 | 1E+08 | ↑ | 8-Mar | 220972 | ↓ |
| ZNF385D | 79750 | ↑ | LRG1 | 116844 | ↑ | EBF4 | 57593 | ↑ | FAM71D | 161142 | ↓ |
| NME5 | 8382 | ↑ | LAG3 | 3902 | ↑ | TMEM238 | 388564 | ↑ | TLR2 | 7097 | ↓ |
| LOC100132832 | 1E+08 | ↑ | APOBEC3H | 164668 | ↑ | DIXDC1 | 85458 | ↑ | ZNF835 | 90485 | ↓ |
| MUC4 | 4585 | ↑ | NACAD | 23148 | ↑ | EFEMP2 | 30008 | ↑ | CXCL12 | 6387 | ↓ |
| GALNT12 | 79695 | ↑ | ZDHHC11 | 79844 | ↑ | CYTH4 | 27128 | ↑ | SNRPG | 6637 | ↓ |
| FGD3 | 89846 | ↑ | AQP5 | 362 | ↑ | ATG16L2 | 89849 | ↑ | TSPAN31 | 6302 | ↓ |
| C14orf182 | 283551 | ↑ | P2RY8 | 286530 | ↑ | ACBD4 | 79777 | ↑ | DUSP4 | 1846 | ↓ |
| RAB33A | 9363 | ↑ | PCDH20 | 64881 | ↑ | CXorf21 | 80231 | ↑ | FERMT1 | 55612 | ↓ |
| PLAT | 5327 | ↑ | C19orf45 | 374877 | ↑ | ST8SIA4 | 7903 | ↑ | APOBEC3C | 27350 | ↓ |
| VSTM2L | 128434 | ↑ | CAMKV | 79012 | ↑ | B4GALNT4 | 338707 | ↑ | ZNF827 | 152485 | ↓ |
| FCGR2A | 2212 | ↑ | LCN15 | 389812 | ↑ | ABCA3 | 21 | ↑ | NBPF24 | 728912 | ↓ |
| PKLR | 5313 | ↑ | RHBDL3 | 162494 | ↑ | TTC28 | 23331 | ↑ | NPB | 256933 | ↓ |
| C1QTNF1 | 114897 | ↑ | PCNXL2 | 80003 | ↑ | CUBN | 8029 | ↑ | CCDC172 | 374355 | ↓ |
| SLC30A3 | 7781 | ↑ | PCDHB9 | 56127 | ↑ | CABP4 | 57010 | ↑ | ZFAT | 57623 | ↓ |
| DRD4 | 1815 | ↑ | KIF21B | 23046 | ↑ | MYLK3 | 91807 | ↑ | TM4SF19 | 116211 | ↓ |
| FGF11 | 2256 | ↑ | LINC00320 | 387486 | ↑ | TMSB15A | 11013 | ↑ | C1QTNF9B-AS1 | 542767 | ↓ |
| TSPAN8 | 7103 | ↑ | PLAC8L1 | 153770 | ↑ | KLC3 | 147700 | ↑ | SLFNL1 | 200172 | ↓ |
| PRRT2 | 112476 | ↑ | TLR1 | 7096 | ↑ | ZMYND10 | 51364 | ↑ | LTB4R2 | 56413 | ↓ |
| LMOD1 | 25802 | ↑ | DNAH7 | 56171 | ↑ | ADC | 113451 | ↑ | RGS5 | 8490 | ↓ |
| MAPT | 4137 | ↑ | LARGE | 9215 | ↑ | NEO1 | 4756 | ↑ | SLITRK2 | 84631 | ↓ |
| MUC5B | 727897 | ↑ | LYPD6B | 130576 | ↑ | THSD1P1 | 374500 | ↑ | DGCR6L | 85359 | ↓ |
| SRCIN1 | 80725 | ↑ | OVGP1 | 5016 | ↑ | REP15 | 387849 | ↑ | AIM1L | 55057 | ↓ |
| MYO1D | 4642 | ↑ | ANKRD20A3 | 441425 | ↑ | LOC219731 | 219731 | ↑ | ABCC2 | 1244 | ↓ |
| C17orf108 | 201229 | ↑ | SRCRB4D | 136853 | ↑ | NLRP11 | 204801 | ↑ | CTSL1P2 | 1517 | ↓ |
| PRTN3 | 5657 | ↑ | AASS | 10157 | ↑ | DHDH | 27294 | ↑ | PCDHB10 | 56126 | ↓ |
| SPTBN4 | 57731 | ↑ | C19orf26 | 255057 | ↑ | ARHGAP44 | 9912 | ↑ | CES4A | 283848 | ↓ |
| TTC9 | 23508 | ↑ | SFTPB | 6439 | ↑ | CCL27 | 10850 | ↑ | ZNF225 | 7768 | ↓ |
| SMPD3 | 55512 | ↑ | ANTXRL | 195977 | ↑ | C20orf196 | 149840 | ↑ | SKP1P2 | 728622 | ↓ |
| STEAP4 | 79689 | ↑ | TGM1 | 7051 | ↑ | PLIN5 | 440503 | ↑ | SNORA80 | 677846 | ↓ |
| NOXRED1 | 122945 | ↑ | NRN1 | 51299 | ↑ | PNPLA5 | 150379 | ↑ | P2RY2 | 5029 | ↓ |
| LOC728743 | 728743 | ↑ | SPON2 | 10417 | ↑ | C2orf72 | 257407 | ↑ | SH2D2A | 9047 | ↓ |
| FLJ34208 | 401106 | ↑ | PRH2 | 5555 | ↑ | SHC3 | 53358 | ↑ | CCNI2 | 645121 | ↓ |
| DOCK6 | 57572 | ↑ | SNORD52 | 26797 | ↑ | EVI5L | 115704 | ↑ | GBX2 | 2637 | ↓ |
| AGAP2 | 116986 | ↑ | C19orf38 | 255809 | ↑ | FAM84B | 157638 | ↑ | C12orf61 | 283416 | ↓ |
| LOC283731 | 283731 | ↑ | FAM99A | 387742 | ↑ | CX3CL1 | 6376 | ↑ | CLIC2 | 1193 | ↓ |
| APBB1IP | 54518 | ↑ | PRDM11 | 56981 | ↑ | HEYL | 26508 | ↑ | OR4E2 | 26686 | ↓ |
| DMD | 1756 | ↑ | SLA2 | 84174 | ↑ | LOC100128822 | 1E+08 | ↑ | POLR2M | 81488 | ↓ |
| LOC386597 | 386597 | ↑ | LINGO1 | 84894 | ↑ | FAM69A | 388650 | ↑ | AURKC | 6795 | ↓ |
| GTF2A1 | 2957 | ↑ | KCNT2 | 343450 | ↑ | PAQR6 | 79957 | ↑ | SYT12 | 91683 | ↓ |
| PLSCR2 | 57047 | ↑ | LOC100130950 | 1E+08 | ↑ | GDF15 | 9518 | ↑ | DHRS2 | 10202 | ↓ |
| BAIAP2L2 | 80115 | ↑ | IL2RB | 3560 | ↑ | RPL32P3 | 132241 | ↑ | TMEM55B | 90809 | ↓ |
| C15orf43 | 145645 | ↑ | TMOD4 | 29765 | ↑ | TMEM198 | 130612 | ↑ | TMEM56-RWDD3 | 1.01E+08 | ↓ |
| B3GNT4 | 79369 | ↑ | ADH4 | 127 | ↑ | NR1H3 | 10062 | ↑ | GATM | 2628 | ↓ |
| PHOSPHO1 | 162466 | ↑ | C2orf82 | 389084 | ↑ | LTB4R | 1241 | ↑ | TNNI2 | 7136 | ↓ |
| C7orf29 | 113763 | ↑ | SLC35G5 | 83650 | ↑ | ORAI3 | 93129 | ↑ | PTGDR | 5729 | ↓ |
| C1orf204 | 284677 | ↑ | COL6A4P2 | 646300 | ↑ | APOBEC3D | 140564 | ↑ | OR10A7 | 121364 | ↓ |
| TREX1 | 11277 | ↑ | C15orf27 | 123591 | ↑ | MAP3K15 | 389840 | ↑ | ACTRT3 | 84517 | ↓ |
| ZNF230 | 7773 | ↑ | LOC286367 | 286367 | ↑ | STOX2 | 56977 | ↑ | PTAFR | 5724 | ↓ |
| DKKL1 | 27120 | ↑ | OLFM4 | 10562 | ↑ | ASB9 | 140462 | ↑ | LIMCH1 | 22998 | ↓ |
| ZNF785 | 146540 | ↑ | C1orf105 | 92346 | ↑ | AGPAT4 | 56895 | ↑ | NME6 | 10201 | ↓ |
| C19orf71 | 1E+08 | ↑ | LYPD6 | 130574 | ↑ | PPP1R16A | 84988 | ↑ | ZNF551 | 90233 | ↓ |
| PODNL1 | 79883 | ↑ | KALRN | 8997 | ↑ | TRPV1 | 7442 | ↑ | MAK | 4117 | ↓ |
| LINC00087 | 644596 | ↑ | C4orf29 | 80167 | ↑ | LOC283070 | 283070 | ↑ | DPY19L2P2 | 349152 | ↓ |
| LEFTY1 | 10637 | ↑ | LOC400655 | 400655 | ↑ | SYT1 | 6857 | ↑ | BDKRB2 | 624 | ↓ |
| VASH2 | 79805 | ↑ | LOC647323 | 647323 | ↑ | FKBP1B | 2281 | ↑ | RPL21P44 | 402176 | ↓ |
| CEACAM19 | 56971 | ↑ | PLB1 | 151056 | ↑ | ANKRD34A | 284615 | ↑ | KRTAP5-2 | 440021 | ↓ |
| C3orf67 | 200844 | ↑ | ATP2B2 | 491 | ↑ | AK4 | 205 | ↑ | IRAK3 | 11213 | ↓ |
| FLJ16779 | 1E+08 | ↑ | NRADDP | 1E+08 | ↑ | ITFG3 | 83986 | ↑ | C2CD3 | 26005 | ↓ |
| MATL2963 | 283314 | ↑ | CYP19A1 | 1588 | ↑ | CCL5 | 6352 | ↑ | PDGFRL | 5157 | ↓ |
| MIR1282 | 1E+08 | ↑ | SEMA3G | 56920 | ↑ | GPR146 | 115330 | ↑ | ZNF169 | 169841 | ↓ |
| SEMA3B | 7869 | ↑ | SERTAD4 | 56256 | ↑ | WBSCR27 | 155368 | ↑ | ZFP37 | 7539 | ↓ |
| B3GALT1 | 8708 | ↑ | STAT4 | 6775 | ↑ | NEU4 | 129807 | ↑ | WFDC3 | 140686 | ↓ |
| LOC100507266 | 1.01E+08 | ↑ | DRP2 | 1821 | ↑ | GCHFR | 2644 | ↑ | BTNL3 | 10917 | ↓ |
| TMEM139 | 135932 | ↑ | LOC284100 | 284100 | ↑ | OLFM2 | 93145 | ↑ | FBF1 | 85302 | ↓ |
| FLJ27352 | 145788 | ↑ | FZD9 | 8326 | ↑ | ZMYM3 | 9203 | ↑ | WT1-AS | 51352 | ↓ |
| IFI27L2 | 83982 | ↑ | SNORD15B | 114599 | ↑ | GTPBP1 | 9567 | ↑ | LRRC37A6P | 387646 | ↓ |
| TSHZ3 | 57616 | ↑ | LOC145820 | 145820 | ↑ | C2CD4C | 126567 | ↑ | FGF1 | 2246 | ↓ |
| TP63 | 8626 | ↑ | DNM1P46 | 196968 | ↑ | GSN | 2934 | ↑ | ABCA13 | 154664 | ↓ |
| CDC42BPG | 55561 | ↑ | ABHD11-AS1 | 171022 | ↑ | RTN2 | 6253 | ↑ | LAMA2 | 3908 | ↓ |
| KLHL30 | 377007 | ↑ | BTN2A3P | 54718 | ↑ | DFNB59 | 494513 | ↑ | GHRLOS2 | 84657 | ↓ |
| OTOF | 9381 | ↑ | CCDC64B | 146439 | ↑ | LZTS2 | 84445 | ↑ | MRAP2 | 112609 | ↓ |
| ABLIM2 | 84448 | ↑ | SLCO2B1 | 11309 | ↑ | IL17RC | 84818 | ↑ | C12orf59 | 120939 | ↓ |
| ATP6V0A4 | 50617 | ↑ | KGFLP2 | 654466 | ↑ | ADAMTS6 | 11174 | ↑ | TLR4 | 7099 | ↓ |
| GMFG | 9535 | ↑ | SERPINF1 | 5176 | ↑ | BNIPL | 149428 | ↑ | GHRLOS | 1E+08 | ↓ |
| SYT5 | 6861 | ↑ | MTMR8 | 55613 | ↑ | LINC00312 | 29931 | ↑ | SLC26A1 | 10861 | ↓ |
| GLI3 | 2737 | ↑ | GPC4 | 2239 | ↑ | ABCC13 | 150000 | ↑ | TEAD4 | 7004 | ↓ |
| SLC2A9 | 56606 | ↑ | SPATA12 | 353324 | ↑ | WASH1 | 1E+08 | ↑ | LOC100188947 | 1E+08 | ↓ |
| TTC25 | 83538 | ↑ | NOVA2 | 4858 | ↑ | SH2B2 | 10603 | ↑ | CPM | 1368 | ↓ |
| LOC283914 | 283914 | ↑ | DPEP1 | 1800 | ↑ | NKAPP1 | 158801 | ↑ | MURC | 347273 | ↓ |
| HSD17B6 | 8630 | ↑ | NRN1L | 123904 | ↑ | RHBDL1 | 9028 | ↑ | AGAP6 | 414189 | ↓ |
| CCDC153 | 283152 | ↑ | LOC440300 | 440300 | ↑ | LHX6 | 26468 | ↑ | GKN1 | 56287 | ↓ |
| FBLN7 | 129804 | ↑ | SH3TC1 | 54436 | ↑ | ZC3H12B | 340554 | ↑ | ZBTB12 | 221527 | ↓ |
| SV2A | 9900 | ↑ | RBM43 | 375287 | ↑ | KNDC1 | 85442 | ↑ | - | 1E+08 | ↓ |
| JAKMIP3 | 282973 | ↑ | NXF3 | 56000 | ↑ | SLC4A11 | 83959 | ↑ | ZNF736 | 728927 | ↓ |
| SNORD18B | 595099 | ↑ | HRNR | 388697 | ↑ | CYP27C1 | 339761 | ↑ | POPDC2 | 64091 | ↓ |
| CYP3A4 | 1576 | ↑ | GRK4 | 2868 | ↑ | CACNA1D | 776 | ↑ | ELAVL2 | 1993 | ↓ |
| DLL1 | 28514 | ↑ | C20orf203 | 284805 | ↑ | HMGN5 | 79366 | ↑ | ZNF597 | 146434 | ↓ |
| CN5H6.4 | 150384 | ↑ | RDH12 | 145226 | ↑ | DNAH12 | 201625 | ↑ | CBX2 | 84733 | ↓ |
| PRR16 | 51334 | ↑ | RGS4 | 5999 | ↑ | LTBP4 | 8425 | ↑ | SSTR5 | 6755 | ↓ |
| PLSCR4 | 57088 | ↑ | GLYCTK | 132158 | ↑ | LOC151174 | 151174 | ↑ | FBXL19-AS1 | 283932 | ↓ |
| PION | 54103 | ↑ | FUT7 | 2529 | ↑ | LTBP3 | 4054 | ↑ | PKHD1 | 5314 | ↓ |
| TMEM221 | 1E+08 | ↑ | FAM66C | 440078 | ↑ | TMEM45A | 55076 | ↑ | INHBA | 3624 | ↓ |
| GDF9 | 2661 | ↑ | OR5AN1 | 390195 | ↑ | BRSK1 | 84446 | ↑ | KCNIP4 | 80333 | ↓ |
| KIR3DL1 | 3811 | ↑ | CACNG1 | 786 | ↑ | FAM118A | 55007 | ↑ | C1QTNF7 | 114905 | ↓ |
| CREB3L3 | 84699 | ↑ | TMEM105 | 284186 | ↑ | CTSH | 1512 | ↑ | CSF3R | 1441 | ↓ |
| PDZD7 | 79955 | ↑ | ATL1 | 51062 | ↑ | NAT6 | 24142 | ↑ | KRT81 | 3887 | ↓ |
| DKFZP434A062 | 26102 | ↑ | TBXAS1 | 6916 | ↑ | SDHAP2 | 727956 | ↑ | HS6ST2 | 90161 | ↓ |
| C14orf132 | 56967 | ↑ | ODF3B | 440836 | ↑ | ADRA2C | 152 | ↑ | MYL5 | 4636 | ↓ |
| AACSP1 | 729522 | ↑ | C5orf47 | 133491 | ↑ | TMTC2 | 160335 | ↑ | FSD1L | 83856 | ↓ |
| ARHGEF37 | 389337 | ↑ | THRA | 7067 | ↑ | SCARNA9 | 619383 | ↑ | FRG2C | 1E+08 | ↓ |
| RTN4RL2 | 349667 | ↑ | FER1L4 | 80307 | ↑ | STAP2 | 55620 | ↑ | HIST1H4D | 8360 | ↓ |
| NEK8 | 284086 | ↑ | MAST3 | 23031 | ↑ | ENGASE | 64772 | ↑ | LOC387723 | 387723 | ↓ |
| ZNF836 | 162962 | ↑ | SERPINA3 | 12 | ↑ | ALDH4A1 | 8659 | ↑ | IL15RA | 3601 | ↓ |
| MR1 | 3140 | ↑ | NEIL1 | 79661 | ↑ | STARD4 | 134429 | ↑ | KIAA0226 | 9711 | ↓ |
| TMEM178 | 130733 | ↑ | RNASE4 | 6038 | ↑ | KCNIP2 | 30819 | ↑ | ANKRD1 | 27063 | ↓ |
| TIMP3 | 7078 | ↑ | PTGES | 9536 | ↑ | HAP1 | 9001 | ↑ | GCSHP3 | 1E+08 | ↓ |
| LPAR3 | 23566 | ↑ | PDK1 | 5163 | ↑ | WHAMMP2 | 440253 | ↑ | LMBRD2 | 92255 | ↓ |
| ARMC12 | 221481 | ↑ | TMEM8B | 51754 | ↑ | EGFL7 | 51162 | ↑ | SOLH | 6650 | ↓ |
| LOC100133957 | 1E+08 | ↑ | PALM | 5064 | ↑ | GRTP1 | 79774 | ↑ | HTR1D | 3352 | ↓ |
| ADAM32 | 203102 | ↑ | ARHGEF25 | 115557 | ↑ | PCYOX1L | 78991 | ↑ | GTF3C4 | 9329 | ↓ |
| COL27A1 | 85301 | ↑ | ACSF2 | 80221 | ↑ | MAPK8IP1 | 9479 | ↑ | DLEU2L | 79469 | ↓ |
| RBM47 | 54502 | ↑ | SBK1 | 388228 | ↑ | FKBP10 | 60681 | ↑ | PANK1 | 53354 | ↓ |
| PLEKHA4 | 57664 | ↑ | ZNF467 | 168544 | ↑ | GRAMD4 | 23151 | ↑ | ZFP112 | 7771 | ↓ |
| GYLTL1B | 120071 | ↑ | AGPHD1 | 123688 | ↑ | SEZ6L2 | 26470 | ↑ | PER3 | 8863 | ↓ |
| MEIS1 | 4211 | ↑ | ALDOC | 230 | ↑ | PTK7 | 5754 | ↑ | PPM1H | 57460 | ↓ |
| POTEM | 641455 | ↑ | TFCP2L1 | 29842 | ↑ | DPYSL4 | 10570 | ↑ | DBIL5P | 1E+08 | ↓ |
| PLEKHG1 | 57480 | ↑ | PLIN4 | 729359 | ↑ | SCNN1D | 6339 | ↑ | ISPD | 729920 | ↓ |
| NLGN3 | 54413 | ↑ | COX6B2 | 125965 | ↑ | GNG4 | 2786 | ↑ | ATF5 | 22809 | ↓ |
| ING4 | 51147 | ↑ | ProSAPiP1 | 9762 | ↑ | ZNF589 | 51385 | ↑ | CLDN1 | 9076 | ↓ |
| RASA2 | 5922 | ↑ | FN3K | 64122 | ↑ | EML6 | 400954 | ↑ | CHST3 | 9469 | ↓ |
| NCRUPAR | 1E+08 | ↑ | GABBR1 | 2550 | ↑ | EPB41 | 2035 | ↑ | FAM84A | 151354 | ↓ |
| IL3RA | 3563 | ↑ | SLC27A1 | 376497 | ↑ | RGS11 | 8786 | ↑ | IRF2BP1 | 26145 | ↓ |
| KCNN1 | 3780 | ↑ | LPAR1 | 1902 | ↑ | SULT4A1 | 25830 | ↑ | C1orf106 | 55765 | ↓ |
| NKX2-8 | 26257 | ↑ | 1-Sep | 1731 | ↑ | LOC146880 | 146880 | ↑ | TMEM107 | 84314 | ↓ |
| HPCA | 3208 | ↑ | SEMA4G | 57715 | ↑ | C16orf74 | 404550 | ↑ | SLC25A5-AS1 | 1E+08 | ↓ |
| MYCL1 | 4610 | ↑ | CXCL3 | 2921 | ↑ | MED12L | 116931 | ↑ | ZNF669 | 79862 | ↓ |
| DNM1P41 | 440299 | ↑ | CERCAM | 51148 | ↑ | SUSD2 | 56241 | ↑ | SPATA13 | 221178 | ↓ |
| MYOM1 | 8736 | ↑ | OSBPL7 | 114881 | ↑ | MRPL42P5 | 359821 | ↑ | FLNC | 2318 | ↓ |
| LINC00239 | 145200 | ↑ | NPR3 | 4883 | ↑ | ZSCAN16 | 80345 | ↑ | SOST | 50964 | ↓ |
| LOC100216001 | 1E+08 | ↑ | PRKCG | 5582 | ↑ | SP6 | 80320 | ↑ | C3orf55 | 152078 | ↓ |
| RASL12 | 51285 | ↑ | GLS2 | 27165 | ↑ | LOC154761 | 154761 | ↑ | ZNF286A | 57335 | ↓ |
| XIST | 7503 | ↑ | EPB41L1 | 2036 | ↑ | NGFR | 4804 | ↑ | MOSPD2 | 158747 | ↓ |
| CRMP1 | 1400 | ↑ | C3orf18 | 51161 | ↑ | GTF2I | 2969 | ↑ | DPY19L2 | 283417 | ↓ |
| HGC6.3 | 1E+08 | ↑ | PFKFB4 | 5210 | ↑ | ARHGAP5-AS1 | 84837 | ↑ | S100A2 | 6273 | ↓ |
| DNAI1 | 27019 | ↑ | FOXO4 | 4303 | ↑ | MAP1A | 4130 | ↑ | PGBD3 | 267004 | ↓ |
| LOC440896 | 440896 | ↑ | ZNF815P | 401303 | ↑ | LAMA4 | 3910 | ↑ | ANXA3 | 306 | ↓ |
| FAM22A | 728118 | ↑ | CD7 | 924 | ↑ | SMAD5-AS1 | 9597 | ↑ | MAPK8 | 5599 | ↓ |
| TMPRSS11BNL | 401136 | ↑ | FLJ22184 | 80164 | ↑ | TEX9 | 374618 | ↑ | GIN1 | 54826 | ↓ |
| C1orf88 | 128344 | ↑ | IL22RA1 | 58985 | ↑ | SYDE2 | 84144 | ↑ | NOL6 | 65083 | ↓ |
| GRID1 | 2894 | ↑ | ZCCHC24 | 219654 | ↑ | PABPC1L2B | 645974 | ↑ | C5orf34 | 375444 | ↓ |
| CD74 | 972 | ↑ | FBXO2 | 26232 | ↑ | TSPAN33 | 340348 | ↑ | RERGL | 79785 | ↓ |
| LDHAL6A | 160287 | ↑ | LDHD | 197257 | ↑ | CHI3L2 | 1117 | ↑ | TMEM170B | 1E+08 | ↓ |
| EFHC2 | 80258 | ↑ | SSC5D | 284297 | ↑ | ZNF778 | 197320 | ↑ | MMP1 | 4312 | ↓ |
| ATG9B | 285973 | ↑ | AMT | 275 | ↑ | AKAP5 | 9495 | ↑ | HSPA4L | 22824 | ↓ |
| KCNK3 | 3777 | ↑ | TM7SF2 | 7108 | ↑ | RNF138P1 | 379013 | ↑ | LOC100131691 | 1E+08 | ↓ |
| CXCL14 | 9547 | ↑ | NOXA1 | 10811 | ↑ | CNTNAP3B | 728577 | ↑ | LENG1 | 79165 | ↓ |
| SALL2 | 6297 | ↑ | BAI1 | 575 | ↑ | PPP1R12B | 4660 | ↑ | RHOBTB2 | 23221 | ↓ |
| DNAJB13 | 374407 | ↑ | CSPG5 | 10675 | ↑ | KLF9 | 687 | ↑ | FAM110A | 83541 | ↓ |
| TMPRSS5 | 80975 | ↑ | LOC388588 | 388588 | ↑ | BCORL1 | 63035 | ↑ | C11orf83 | 790955 | ↓ |
| RIBC2 | 26150 | ↑ | CPS1 | 1373 | ↑ | CCBL1 | 883 | ↑ | LOC643837 | 643837 | ↓ |
| RAB39A | 54734 | ↑ | BCAS3 | 54828 | ↑ | IER5L | 389792 | ↑ | TIRAP | 114609 | ↓ |
| SAA1 | 6288 | ↑ | LOC100507421 | 1.01E+08 | ↑ | LRRC26 | 389816 | ↑ | NUPL2 | 11097 | ↓ |
| RAC2 | 5880 | ↑ | FCHSD1 | 89848 | ↑ | TMC6 | 11322 | ↑ | MATN2 | 4147 | ↓ |
| SFRP5 | 6425 | ↑ | SLC41A2 | 84102 | ↑ | SLC26A6 | 65010 | ↑ | ZNF35 | 7584 | ↓ |
| SLC52A1 | 55065 | ↑ | CEACAM22P | 388550 | ↑ | SLC25A45 | 283130 | ↑ | ATF2 | 1386 | ↓ |
| CCDC108 | 255101 | ↑ | RAB3A | 5864 | ↑ | HSPA2 | 3306 | ↑ | EGFLAM | 133584 | ↓ |
| ZNF93 | 81931 | ↑ | RTBDN | 83546 | ↑ | PFKL | 5211 | ↑ | PPP4R2 | 151987 | ↓ |
| MMEL1 | 79258 | ↑ | TMEM52 | 339456 | ↑ | SDK1 | 221935 | ↑ | SNAR-E | 1E+08 | ↓ |
| WDR65 | 149465 | ↑ | APBB1 | 322 | ↑ | FAM132A | 388581 | ↑ | LOC644961 | 644961 | ↓ |
| ELOVL3 | 83401 | ↑ | RAB26 | 25837 | ↑ | ZNF615 | 284370 | ↑ | ZNF782 | 158431 | ↓ |
| PTGER3 | 5733 | ↑ | PIGZ | 80235 | ↑ | JHDM1D | 80853 | ↑ | EOMES | 8320 | ↓ |
| MYOM2 | 9172 | ↑ | GDPD5 | 81544 | ↑ | ZNF219 | 51222 | ↑ | LOC100132707 | 1E+08 | ↓ |
| GAL3ST4 | 79690 | ↑ | OPRL1 | 4987 | ↑ | RNF123 | 63891 | ↑ | MAFF | 23764 | ↓ |
| NTRK1 | 4914 | ↑ | ERBB3 | 2065 | ↑ | ROGDI | 79641 | ↑ | FLJ39534 | 285352 | ↓ |
| ILDR2 | 387597 | ↑ | CAPS2 | 84698 | ↑ | NUPR1 | 26471 | ↑ | ID2 | 3398 | ↓ |
| FAM183A | 440585 | ↑ | ZNF117 | 51351 | ↑ | C7orf57 | 136288 | ↑ | ZNF777 | 27153 | ↓ |
| C1orf170 | 84808 | ↑ | FAM69B | 138311 | ↑ | TSPAN18 | 90139 | ↑ | C12orf45 | 121053 | ↓ |
| MFSD2A | 84879 | ↑ | SERGEF | 26297 | ↑ | RGL3 | 57139 | ↑ | PBX3 | 5090 | ↓ |
| PPP1R3G | 648791 | ↑ | OBSCN | 84033 | ↑ | GAS6 | 2621 | ↑ | ENOX2 | 10495 | ↓ |
| LRRC29 | 26231 | ↑ | INSR | 3643 | ↑ | ITPR1 | 3708 | ↑ | LHFP | 10186 | ↓ |
| KIAA0125 | 9834 | ↑ | LOC728730 | 728730 | ↑ | TNK2 | 10188 | ↑ | KIAA2018 | 205717 | ↓ |
| SDR16C5 | 195814 | ↑ | H19 | 283120 | ↑ | WDR17 | 116966 | ↑ | C12orf43 | 64897 | ↓ |
| ELANE | 1991 | ↑ | PAQR8 | 85315 | ↑ | FANK1 | 92565 | ↑ | NAV3 | 89795 | ↓ |
| EMR4P | 326342 | ↑ | TLE6 | 79816 | ↑ | IFITM1 | 8519 | ↑ | EFNB1 | 1947 | ↓ |
| ASGR1 | 432 | ↑ | NES | 10763 | ↑ | FAM71E1 | 112703 | ↑ | ERCC6L2 | 375748 | ↓ |
| IL12A | 3592 | ↑ | ELF3 | 1999 | ↑ | UPK1B | 7348 | ↑ | CCNJ | 54619 | ↓ |
| SH2D3C | 10044 | ↑ | GSTO2 | 119391 | ↑ | LYPD5 | 284348 | ↑ | THBS1 | 7057 | ↓ |
| LOC441454 | 441454 | ↑ | HDAC5 | 10014 | ↑ | LOC144486 | 144486 | ↑ | CPNE4 | 131034 | ↓ |
| ATP2C2 | 9914 | ↑ | FAM131C | 348487 | ↑ | HLA-J | 3137 | ↑ | XRCC2 | 7516 | ↓ |
| BTBD16 | 118663 | ↑ | PBXIP1 | 57326 | ↑ | MT1F | 4494 | ↑ | UBE3D | 90025 | ↓ |
| LOC644242 | 644242 | ↑ | MORC2-AS1 | 150291 | ↑ | TSPYL2 | 64061 | ↑ | ZNF57 | 126295 | ↓ |
| RDH16 | 8608 | ↑ | PDE4D | 5144 | ↑ | DYRK1B | 9149 | ↑ | PIGW | 284098 | ↓ |
| PTGER4P2 | 442421 | ↑ | PLA2G6 | 8398 | ↑ | MRC2 | 9902 | ↑ | RQCD1 | 9125 | ↓ |
| MME | 4311 | ↑ | CLDND2 | 125875 | ↑ | CRAT | 1384 | ↑ | ZNF678 | 339500 | ↓ |
| MECOM | 2122 | ↑ | XAF1 | 54739 | ↑ | SLC5A1 | 6523 | ↑ | RCBTB2 | 1102 | ↓ |
| CNTD2 | 79935 | ↑ | ALDH6A1 | 4329 | ↑ | GLRX | 2745 | ↑ | LOC100009676 | 1E+08 | ↓ |
| FUT10 | 84750 | ↑ | TBC1D16 | 125058 | ↑ | UNC93B1 | 81622 | ↑ | AIM1 | 202 | ↓ |
| C1orf21 | 81563 | ↑ | TRIM9 | 114088 | ↑ | XYLT2 | 64132 | ↑ | PCA3 | 50652 | ↓ |
| FITM1 | 161247 | ↑ | MPP2 | 4355 | ↑ | RIMKLA | 284716 | ↑ | ZNF397 | 84307 | ↓ |
| SLC44A3 | 126969 | ↑ | CA11 | 770 | ↑ | AKT3 | 10000 | ↑ | KCNT1 | 57582 | ↓ |
| EPB41L4A | 64097 | ↑ | TSC22D3 | 1831 | ↑ | ADM2 | 79924 | ↑ | PMFBP1 | 83449 | ↓ |
| MMP25 | 64386 | ↑ | SLC45A1 | 50651 | ↑ | TMEM121 | 80757 | ↑ | GLMN | 11146 | ↓ |
| A1CF | 29974 | ↑ | DTNB | 1838 | ↑ | FAM13A-AS1 | 285512 | ↑ | MOCS3 | 27304 | ↓ |
| FAM110C | 642273 | ↑ | HOOK2 | 29911 | ↑ | DNMT3A | 1788 | ↑ | SNHG8 | 1E+08 | ↓ |
| POU2F2 | 5452 | ↑ | TP53INP2 | 58476 | ↑ | ANKRD36B | 57730 | ↑ | CASC5 | 57082 | ↓ |
| ADAMTS18 | 170692 | ↑ | AIF1L | 83543 | ↑ | SLC2A1 | 6513 | ↑ | ADAMTS4 | 9507 | ↓ |
| EFCAB9 | 285588 | ↑ | CREB3L2 | 64764 | ↑ | C19orf66 | 55337 | ↑ | S100A16 | 140576 | ↓ |
| CDHR4 | 389118 | ↑ | AZU1 | 566 | ↑ | JUND | 3727 | ↑ | ANKRD6 | 22881 | ↓ |
| PKD1L1 | 168507 | ↑ | BNIP3 | 664 | ↑ | PID1 | 55022 | ↑ | GCLM | 2730 | ↓ |
| TJP3 | 27134 | ↑ | ATP6V1G2 | 534 | ↑ | C14orf28 | 122525 | ↑ | TBC1D30 | 23329 | ↓ |
| CYP39A1 | 51302 | ↑ | ATP2A3 | 489 | ↑ | SEMA3F | 6405 | ↑ | ACVR2A | 92 | ↓ |
| CBLN3 | 643866 | ↑ | FCHO1 | 23149 | ↑ | WIPF1 | 7456 | ↑ | SUN3 | 256979 | ↓ |
| ICAM4 | 3386 | ↑ | DAPK2 | 23604 | ↑ | MPI | 4351 | ↑ | ARL5B | 221079 | ↓ |
| MIR1538 | 1E+08 | ↑ | LHPP | 64077 | ↑ | GATSL3 | 652968 | ↑ | ZBTB49 | 166793 | ↓ |
| LOC440910 | 440910 | ↑ | LOC283335 | 283335 | ↑ | PKDCC | 91461 | ↑ | LINC00256A | 286333 | ↓ |
| SNAP91 | 9892 | ↑ | SLC18B1 | 116843 | ↑ | BTN2A2 | 10385 | ↑ | CDKN2AIP | 55602 | ↓ |
| AK8 | 158067 | ↑ | MAGIX | 79917 | ↑ | MOSPD3 | 64598 | ↑ | ATF3 | 467 | ↓ |
| SNORA21 | 619505 | ↑ | LYPD3 | 27076 | ↑ | ADRA1B | 147 | ↑ | NUDT16P1 | 152195 | ↓ |
| ADRA1D | 146 | ↑ | MAGEA12 | 4111 | ↑ | OPN1SW | 611 | ↑ | ANKRD36 | 375248 | ↓ |
| POLN | 353497 | ↑ | ASIC1 | 41 | ↑ | ALDH5A1 | 7915 | ↑ | TXNRD3 | 114112 | ↓ |
| FOXD2 | 2306 | ↑ | PTP4A3 | 11156 | ↑ | SMAD6 | 4091 | ↑ | APITD1 | 378708 | ↓ |
| APOD | 347 | ↑ | MXRA5 | 25878 | ↑ | SLC6A20 | 54716 | ↑ | KLLN | 1E+08 | ↓ |
| GAL3ST1 | 9514 | ↑ | LOC100506835 | 1.01E+08 | ↑ | SNCB | 6620 | ↑ | GAS2L3 | 283431 | ↓ |
| BEX4 | 56271 | ↑ | TTLL1 | 25809 | ↑ | NFASC | 23114 | ↑ | NINJ1 | 4814 | ↓ |
| IL23A | 51561 | ↑ | IGFBP3 | 3486 | ↑ | HERC3 | 8916 | ↑ | MARS2 | 92935 | ↓ |
| NHLH1 | 4807 | ↑ | RHOBTB1 | 9886 | ↑ | MEIG1 | 644890 | ↑ | CDKN2D | 1032 | ↓ |
| LOC285103 | 285103 | ↑ | DGKA | 1606 | ↑ | TEAD3 | 7005 | ↑ | SERPINB8 | 5271 | ↓ |
| CASP10 | 843 | ↑ | SLITRK6 | 84189 | ↑ | VIT | 5212 | ↑ | BFSP1 | 631 | ↓ |
| DCST2 | 127579 | ↑ | FADS2 | 9415 | ↑ | HS3ST6 | 64711 | ↑ | GIT1 | 28964 | ↓ |
| ABCA11P | 79963 | ↑ | VWA5A | 4013 | ↑ | EML2 | 24139 | ↑ | LAMC2 | 3918 | ↓ |
| MAPK10 | 5602 | ↑ | TBX18 | 9096 | ↑ | DYNC2LI1 | 51626 | ↑ | CCDC75 | 253635 | ↓ |
| LINC00114 | 400866 | ↑ | DIRAS1 | 148252 | ↑ | LPPR3 | 79948 | ↑ | OR7D2 | 162998 | ↓ |
| MIR210HG | 1.01E+08 | ↑ | FGFR4 | 2264 | ↑ | JUNB | 3726 | ↑ | ITGA8 | 8516 | ↓ |
| DDIT4 | 54541 | ↑ | GNAZ | 2781 | ↑ | ACVR1B | 91 | ↑ | NOC3L | 64318 | ↓ |
| ESRP1 | 54845 | ↑ | ZNF204P | 7754 | ↑ | AP1G2 | 8906 | ↑ | PCYT1A | 5130 | ↓ |
| LOC100190940 | 1E+08 | ↑ | RWDD2A | 112611 | ↑ | DPM3 | 54344 | ↑ | ZNF304 | 57343 | ↓ |
| ICAM5 | 7087 | ↑ | HTRA1 | 5654 | ↑ | NISCH | 11188 | ↑ | C1orf56 | 54964 | ↓ |
| SCARNA12 | 677777 | ↑ | PLEKHG6 | 55200 | ↑ | PRDX2 | 7001 | ↑ | GVINP1 | 387751 | ↓ |
| C17orf107 | 1E+08 | ↑ | PIPOX | 51268 | ↑ | PLOD1 | 5351 | ↑ | IL1RAP | 3556 | ↓ |
| SLC13A4 | 26266 | ↑ | DOPEY2 | 9980 | ↑ | TNIK | 23043 | ↑ | ELMO2 | 63916 | ↓ |
| PRODH | 5625 | ↑ | HOXA5 | 3202 | ↑ | AMDHD1 | 144193 | ↑ | STXBP5 | 134957 | ↓ |
| LOC100132215 | 1E+08 | ↑ | APOBEC3G | 60489 | ↑ | SCAMP5 | 192683 | ↑ | DBF4 | 10926 | ↓ |
| LOC100506939 | 1.01E+08 | ↑ | ARAP3 | 64411 | ↑ | CLIP2 | 7461 | ↑ | CCDC103 | 388389 | ↓ |
| PSD2 | 84249 | ↑ | CCDC68 | 80323 | ↑ | FCGRT | 2217 | ↑ | WDR83 | 84292 | ↓ |
| LTK | 4058 | ↑ | ANGPTL4 | 51129 | ↑ | TRPA1 | 8989 | ↑ | IDH3B | 3420 | ↓ |
| DDC | 1644 | ↑ | TCEA2 | 6919 | ↑ | ABCA2 | 20 | ↑ | OR51G2 | 81282 | ↓ |
| MTUS2 | 23281 | ↑ | GUCA1B | 2979 | ↑ | LOC100129148 | 1E+08 | ↑ | LPXN | 9404 | ↓ |
| SALL4 | 57167 | ↑ | LRRC48 | 83450 | ↑ | MXRA8 | 54587 | ↑ | SIAE | 54414 | ↓ |
| KLRC3 | 3823 | ↑ | CXCL16 | 58191 | ↑ | CRELD1 | 78987 | ↑ | DUSP18 | 150290 | ↓ |
| LOC100616668 | 1.01E+08 | ↑ | HILPDA | 29923 | ↑ | 2-Mar | 51257 | ↑ | SRRD | 402055 | ↓ |
| C2orf89 | 129293 | ↑ | CLEC2D | 29121 | ↑ | PANX2 | 56666 | ↑ | C17orf62 | 79415 | ↓ |
| WDR90 | 197335 | ↑ | OSBPL5 | 114879 | ↑ | GAB1 | 2549 | ↑ | BBOX1 | 8424 | ↓ |
| ROBO2 | 6092 | ↑ | PDK3 | 5165 | ↑ | ADA | 100 | ↑ | LOC100129196 | 1E+08 | ↓ |
| DUSP15 | 128853 | ↑ | ACAP1 | 9744 | ↑ | C2orf81 | 388963 | ↑ | PUS7L | 83448 | ↓ |
| CPNE9 | 151835 | ↑ | KCNH2 | 3757 | ↑ | MAPK3 | 5595 | ↑ | AA06 | 1.01E+08 | ↓ |
| CHST5 | 23563 | ↑ | JAK3 | 3718 | ↑ | ATHL1 | 80162 | ↑ | MYO3B | 140469 | ↓ |
| GOLGA8IP | 283796 | ↑ | CTHRC1 | 115908 | ↑ | MKS1 | 54903 | ↑ | ZNF142 | 7701 | ↓ |
| IGF2-AS | 51214 | ↑ | GSTA4 | 2941 | ↑ | ADAMTS10 | 81794 | ↑ | FAM151B | 167555 | ↓ |
| CDK14 | 5218 | ↑ | IMMP2L | 83943 | ↑ | CRTC1 | 23373 | ↑ | SAMHD1 | 25939 | ↓ |
| CERKL | 375298 | ↑ | ALDH3B1 | 221 | ↑ | CITED4 | 163732 | ↑ | CHCHD4 | 131474 | ↓ |
| MMP11 | 4320 | ↑ | PADI2 | 11240 | ↑ | PPFIA3 | 8541 | ↑ | TAF4B | 6875 | ↓ |
| NHLRC4 | 283948 | ↑ | PLLP | 51090 | ↑ | DHRS13 | 147015 | ↑ | ORC1 | 4998 | ↓ |
| KIAA1984 | 84960 | ↑ | RASSF4 | 83937 | ↑ | PLCXD1 | 55344 | ↑ | LOC642361 | 642361 | ↓ |
| HYAL1 | 3373 | ↑ | ARHGEF40 | 55701 | ↑ | LOC100127888 | 1E+08 | ↑ | STAB1 | 23166 | ↓ |
| CDSN | 1041 | ↑ | H6PD | 9563 | ↑ | SAMD9 | 54809 | ↑ | LOC340515 | 340515 | ↓ |
| MAP2K6 | 5608 | ↑ | SLC2A12 | 154091 | ↑ | MAP1LC3A | 84557 | ↑ | EMG1 | 10436 | ↓ |
| BK250D10.8 | 339674 | ↑ | LPAR6 | 10161 | ↑ | ST6GALNAC6 | 30815 | ↑ | XKR8 | 55113 | ↓ |
| TNFSF10 | 8743 | ↑ | ANKRD24 | 170961 | ↑ | CD24 | 1E+08 | ↑ | ANKRD33B | 651746 | ↓ |
| ERCC5 | 2073 | ↑ | KANK3 | 256949 | ↑ | OBSL1 | 23363 | ↑ | DCLRE1C | 64421 | ↓ |
| LOC152024 | 152024 | ↑ | CDC42EP5 | 148170 | ↑ | CDKN1A | 1026 | ↑ | NDUFA7 | 4701 | ↓ |
| SNORD112 | 692215 | ↑ | TCTN2 | 79867 | ↑ | PKD1 | 5310 | ↑ | ZNF101 | 94039 | ↓ |
| LOC100233209 | 1E+08 | ↑ | TNNI3 | 7137 | ↑ | BIRC7 | 79444 | ↑ |  |  |  |

Table S5. Functional categories of common differentially expressed genes (DEGs) in PANC-1 cells of overexpressing MUC4/Y as compared with both control cell lines

| **Subdirectory of GO** | **Accession** | **Gene Ontology term** | **Cluster frequency** | **Corrected *P*-value** | **General description** |
| --- | --- | --- | --- | --- | --- |
| Cellular Component | GO:0042995 | cell projection | 106 out of 1177 genes, 9.0% | 0.0000386 | A prolongation or process extending from a cell, e.g. a flagellum or axon. |
|  | GO:004300 | neuron projection | 60 out of 1177 genes, 5.1% | 0.00213 | A prolongation or process extending from a nerve cell, e.g. an axon or dendrite. |
|  | GO:0016020 | membrane | 584 out of 1177 genes, 49.6% | 0.00303 | Double layer of lipid molecules that encloses all cells, and, in eukaryotes, many organelles; may be a single or double lipid bilayer; also includes associated proteins. |
|  | GO:0005576 | extracellular region | 116 out of 1177 genes, 9.9% | 0.00941 | The space external to the outermost structure of a cell. |
|  | GO:0016021 | integral to membrane | 144 out of 1177 genes, 12.2% | 0.01163 | Penetrating at least one phospholipid bilayer of a membrane. May also refer to the state of being buried in the bilayer with no exposure outside the bilayer. When used to describe a protein, indicates that all or part of the peptide sequence is embedded in the membrane. |
|  | GO:0044421 | extracellular region part | 113 out of 1177 genes, 9.6% | 0.01597 | The space external to the outermost structure of a cell. |
|  | GO:0044425 | membrane part | 489 out of 1177 genes, 41.5% | 0.02197 | Any constituent part of a membrane, a double layer of lipid molecules that encloses all cells, and, in eukaryotes, many organelles; may be a single or double lipid bilayer; also includes associated proteins. |
|  | GO:0031224 | intrinsic to membrane | 434 out of 1177 genes, 36.9% | 0.02439 | Located in a membrane such that some covalently attached portion of the gene product, for example part of a peptide sequence or some other covalently attached group such as a GPI anchor, spans or is embedded in one or both leaflets of the membrane. |
| Molecular Function | GO:0005125 | cytokine activity | 13 out of 1126 genes, 1.2% | 0.02157 | Functions to control the survival, growth, differentiation and effector function of tissues and cells. |
|  | GO:0001530 | lipopolysaccharide binding | 4 out of 1126 genes, 0.4% | 0.04665 | Interacting selectively and non-covalently with lipopolysaccharide. |
| Biological Process | GO:0023052 | signaling | 368 out of 1071 genes, 34.4% | 0.0000283 | The entirety of a process in which information is transmitted within a biological system. This process begins with an active signal and ends when a cellular response has been triggered. |
|  | GO:0023060 | signal transmission | 283 out of 1071 genes, 26.4% | 0.0000599 | The process in which a signal is released and/or conveyed from one location to another. |
|  | GO:0023052 | signaling process | 283 out of 1071 genes, 26.4% | 0.0000779 | The entirety of a process in which information is transmitted within a biological system. This process begins with an active signal and ends when a cellular response has been triggered. |
|  | GO:0007154 | cell communication | 87 out of 1071 genes, 8.1% | 0.0303 | Any process that mediates interactions between a cell and its surroundings. Encompasses interactions such as signaling or attachment between one cell and another cell, between a cell and an extracellular matrix, or between a cell and any other aspect of its environment. |
| DEGs were annotated according to the indicated Gene Ontology system. *P*-values indicate the statistical significance of the enrichment of these terms as calculated by Fisher’s exact test and corrected for multiple testing using the Bonferroni method, *P* ≤0.05. | | | | | |

Table S6. Representative KEGG pathways from signaling pathway impact analysis of DEGs in PANC-1 cells of over-expressing MUC4/Y compared with both control cell lines. DEGs were annotated with the indicated KEGG database

| **Pathway ID** | **Pathway** | **DEGs with pathway annotation (1247)** | **Corrected *P*-value** | **Predicted status in PANC-1- MUC4/Y cell** | **relationship with cancer and Ref.** | **status in MUC4 and Ref.** |
| --- | --- | --- | --- | --- | --- | --- |
| ko04010 | MAPK signaling pathway | 57 (4.57%) | 0.004795669 | Activated | Abnormalities in MAPK signalling play a critical role in the development and progression of cancer [47-49] | Role of MUC4 in cancer cell signaling mediated by the downstream effector molecules, including Ras, Raf, mitogenactivated protein kinase (MAPK), and so on [7] |
| ko04062 | Chemokine signaling pathway | 42 (3.37%) | 0.027943711 | Activated | Chemokine signaling in cancer: Implications on the tumor microenvironment and therapeutic targeting [48-50] | IL-4,9 induced MUC4 enhancement [51,52] |
| ko04060 | Cytokine-cytokine receptor interaction | 41 (3.29%) | 0.027943711 | Activated | cytokine receptor signaling are a hallmark of malignant tumors [48,49,53] | Cytokines induced MUC4 enhancement and MUC4-expressing pancreatic adenocarcinomas show elevated levels of both T1 and T2 cytokines [51,52,54] |
| ko04350 | TGF-beta signaling pathway | 23 (1.84%) | 0.047448396 | Activated | TGF-beta and its signaling effectors act as key determinants of carcinoma cell behavior [55-57] | TGF-beta regulated the role of MUC4-ErbB2 in pancreatic carcinogenesis [58] |
| *P*-values were FDR-corrected for multiple testing, *P* ≤ 0.05. Status predictions were obtained by signaling pathway impact analysis [46], taking fold-change estimates and pathway topology into account. KEGG, Kyoto Encyclopedia of Genes and Genomes; DEG, differentially expressed gene; FDR, False Discovery Rate, MAPK, mitogen-activated protein kinase; TGF, transforming growth factor. | | | | | | |
